# Multivalent Viral Capsids with Internal Cargo for Fibrin Imaging

**DOI:** 10.1371/journal.pone.0100678

**Published:** 2014-06-24

**Authors:** Allie C. Obermeyer, Stacy L. Capehart, John B. Jarman, Matthew B. Francis

**Affiliations:** 1 Department of Chemistry, University of California, Berkeley, California, United States of America; 2 Materials Sciences Division, Lawrence Berkeley National Laboratories, Berkeley, California, United States of America; Oak Ridge National Laboratory, United States of America

## Abstract

Thrombosis is the cause of many cardiovascular syndromes and is a significant contributor to life-threatening diseases, such as myocardial infarction and stroke. Thrombus targeted imaging agents have the capability to provide molecular information about pathological clots, potentially improving detection, risk stratification, and therapy of thrombosis-related diseases. Nanocarriers are a promising platform for the development of molecular imaging agents as they can be modified to have external targeting ligands and internal functional cargo. In this work, we report the synthesis and use of chemically functionalized bacteriophage MS2 capsids as biomolecule-based nanoparticles for fibrin imaging. The capsids were modified using an oxidative coupling reaction, conjugating ∼90 copies of a fibrin targeting peptide to the exterior of each protein shell. The ability of the multivalent, targeted capsids to bind fibrin was first demonstrated by determining the impact on thrombin-mediated clot formation. The modified capsids out-performed the free peptides and were shown to inhibit clot formation at effective concentrations over ten-fold lower than the monomeric peptide alone. The installation of near-infrared fluorophores on the interior surface of the capsids enabled optical detection of binding to fibrin clots. The targeted capsids bound to fibrin, exhibiting higher signal-to-background than control, non-targeted MS2-based nanoagents. The in vitro assessment of the capsids suggests that fibrin-targeted MS2 capsids could be used as delivery agents to thrombi for diagnostic or therapeutic applications.

## Introduction

Cardiovascular disease is the leading cause of mortality in the United States, currently accounting for one in three deaths overall [1]. A significant contributor to the most life-threatening cardiovascular diseases is thrombosis, which is the formation of blood clots within the vascular system. These clots can, in turn, occlude blood vessels and lead to ischemic syndromes including myocardial infarction, stroke, pulmonary embolism, and deep vein thrombosis. The noninvasive identification and molecular characterization of thrombi could lead to improved detection of many of these conditions, and could provide a useful means to monitor the effectiveness of different treatment methods [2], [3], [4], [5], [6]. A set of targeted agents that can specifically associate with clots for the purpose of delivering PET radiotracers, MRI contrast enhancement agents, or long wavelength responsive chromophores is needed.

Nanomaterials could have several distinct advantages for the targeting and imaging of molecular markers of thrombosis. Chief among them is their large surface area, relative to small molecule diagnostic agents, which could allow for the presentation of multiple copies of targeting groups for enhanced binding avidity. Nanoscale carriers can also be tailored to house multiple copies of the reporter moieties, allowing greater signal-to-noise ratios to be achieved. With these concepts in mind, many synthetic nanoparticles have been developed for the purpose of targeted imaging [7], including synthetic polymers [8], liposomes [9], dendrimers [10], [11], and inorganic nanoparticles [12], [13]. Another approach to preparing nanocarrier platforms relies on functionalized biomolecular assemblies, such as viruses and virus-like particles (VLPs) [14], [15], [16], [17], as well as mammalian protein cages including heat shock cages [18], ferritins [19], [20], [21], and vaults [22]. These protein-based nanoparticles have the natural advantage of being monodisperse, nontoxic, and biodegradable.

Recent work has shown the utility of biomolecular assemblies, such as VLPs, for targeted delivery to cancer [23], [24], [25] and sites of inflammation [14], [18], [19], [26]. In these examples, target binding peptide sequences have been incorporated directly into the protein monomers [18], or synthetic targeting agents have been installed using chemical bioconjugation techniques [25]. Although both strategies can be quite effective, the latter approach has the advantage of increased modularity, and it can be used with virtually any targeting agent, including aptamers [27], [28], peptoids [29], [30], and engineered protein binders [31], [32]. Our group has previously used this strategy to functionalize bacteriophage MS2 VLPs for targeted delivery [24].

Prior work on MS2-based delivery agents has shown that MS2 capsids can be heterologously expressed in *E. coli*, where they self-assemble from 180 sequence-identical monomers to form 27 nm diameter icosahedral capsids. These hollow MS2 capsids are stable toward a wide range of temperature, pH, and ionic strength conditions, and are amenable to genetic mutation and sequence insertions [33], [34], [35]. The assembled capsids contain 32 pores that permit the diffusion of small molecules (< 2 nm) to the interior of the VLPs. These pores, in conjunction with the mutation of a residue on the interior surface of the capsid to a uniquely reactive cysteine, have been used for the conjugation of up to 180 maleimide-functionalized cargo molecules inside each carrier [24]. The subsequent attachment of targeting groups to the exterior surface has been accomplished with high efficiency by targeting an unnatural amino acid, *p*-aminophenylalanine (*p*AF) [34]. The side chain aniline group of this amino acid can be modified with *o*-aminophenol-containing targeting groups using a periodate-mediated oxidative coupling [36]. Other strategies for preparing targeted MS2-based carriers have relied on the attachment of synthetic peptides to exterior lysine residues [25], [37].

Herein, we describe the use of this oxidative coupling strategy to synthesize bacteriophage MS2-based VLPs that are conjugated to synthetic peptide targeting groups. These structures also contain multiple copies of near-infrared optical dyes for facile detection in imaging experiments. The modified capsids were found to inhibit fibrin clot formation at a concentration significantly lower than the free peptide, demonstrating the benefits of using multivalent nanoscale structures for target binding. Additionally, functionalized MS2 capsids were used for the *in vitro* optical imaging of fibrin clots. This *in vitro* work demonstrates the potential of MS2 VLPs for targeted molecular imaging of cardiovascular disease, setting the stage for subsequent *in vivo* studies.

## Results and Discussion

In this work, a peptide targeting group (GPR) with an affinity for fibrin was chosen to create a thrombus targeted VLP [38]. The GPR peptide was derived from the N-terminus of the α-chain of fibrin (Gly-Pro-Arg), and was discovered to bind to a pocket in the C-terminal region of the γ-chain [39], [40]. The originally discovered peptide was shown to inhibit thrombin-mediated fibrin clotting by competitively binding to the fibrin polymerization pocket. Subsequent optimization of the original peptide has identified an extended peptide with improved fibrin-binding and increased proteolytic resistance (Gly-Pro-Arg-Pro-Pro) [41]. This updated peptide has been used to image pulmonary emboli in swine and more recently has been attached to cross-linked iron oxide particles for the *ex vivo* imaging of intravascular thrombi in mice using fluorescence reflectance imaging [42], [43]. Although a variety of other thrombus targeting strategies have proven effective and could likely be used with similar results, the GPR-based targeting strategy was chosen because this peptide has been used successfully for *in vivo* imaging of thrombi [42], [43], [44], [45], [46], [47].

**Figure 1:**
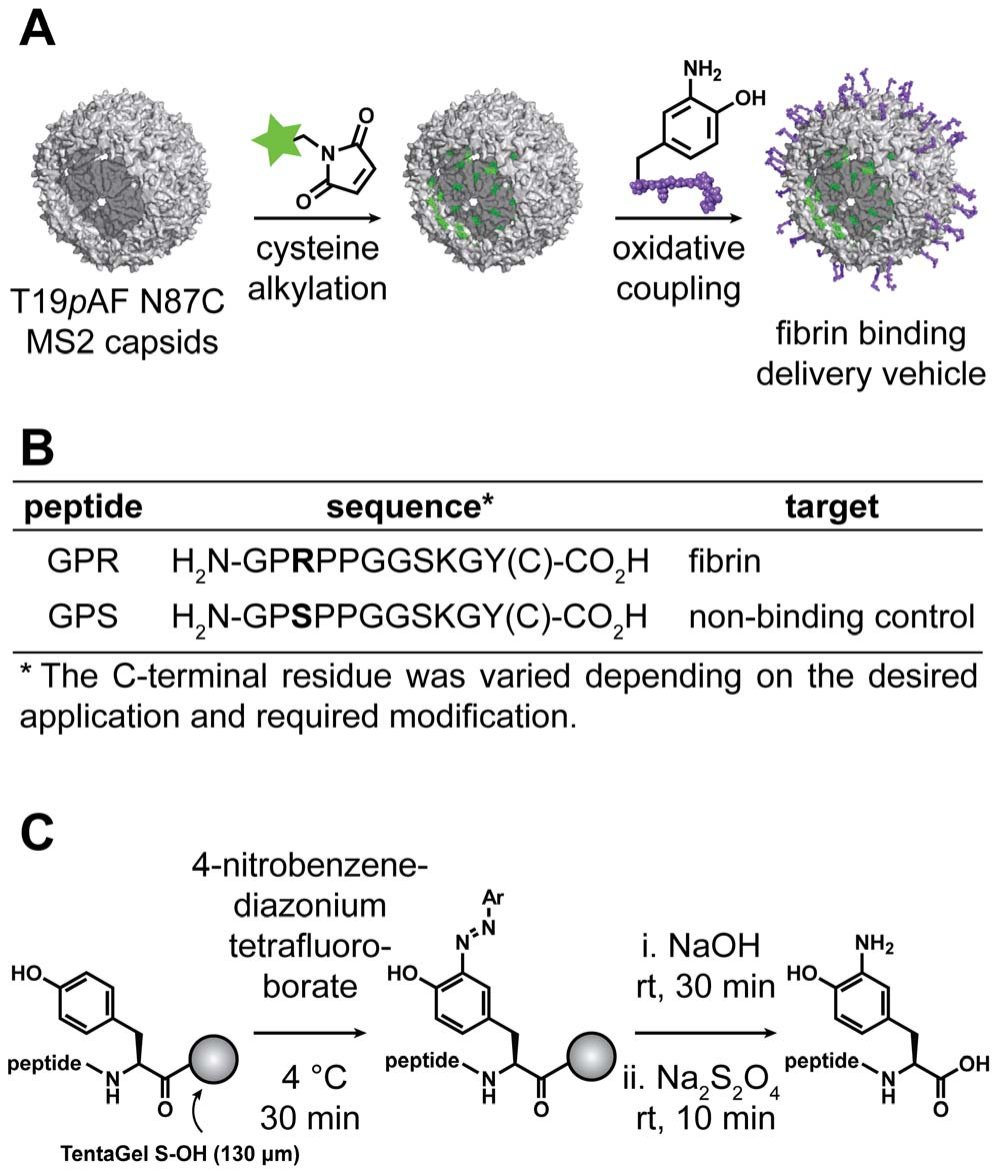
Design of a viral capsid-based targeting system. (a) A scheme is shown for the synthesis of multivalent viral capsids for fibrin imaging. (b) Peptides used for attachment to MS2 or binding to fibrin. Peptides with a cysteine at the C-terminus were modified with a fluorescent dye and used for fibrin binding, while peptides with a C-terminal tyrosine were coupled to MS2 and tested for fibrin clotting inhibition. (c) Scheme for *o*-aminophenol peptide synthesis. The C-terminal tyrosine residue was coupled to 4-nitrobenzenediazonium tetrafluoroborate. The azo peptide was then cleaved from the resin and reduced to the *o*-aminophenol by sodium dithionite.

The scheme for the synthesis of the fibrin targeted delivery vehicle consisted of the selective modification of the interior of MS2 with optical imaging agents, followed by the coupling of the targeting peptide to the exterior surface (Figure 1a). A GPR peptide bearing an *o*-aminophenol for coupling to MS2 was prepared using solid phase peptide synthesis (Figure 1b) [48]. The sequence Gly-Gly-Ser-Lys-Gly-Tyr was added to the peptide to increase the spacing between the binding residues and the capsid, as well as provide a functional handle for modification (Tyr). For peptide samples used in optical imaging experiments, a cysteine residue was also included at the C-terminus to allow modification with the same near-infrared maleimide dye that was introduced in the VLPs (see below). This allowed the binding behavior of the free peptides and the peptide-MS2 conjugates to be compared directly. A non-binding peptide analog was also synthesized as a negative control. It has been reported that a single amino acid change in the GPR peptide (Arg to Ser) drastically decreases its affinity for fibrin [38]. This peptide (GPS) was used as a negative control both as a free peptide and after conjugation to MS2 (GPS-MS2).

**Figure 2:**
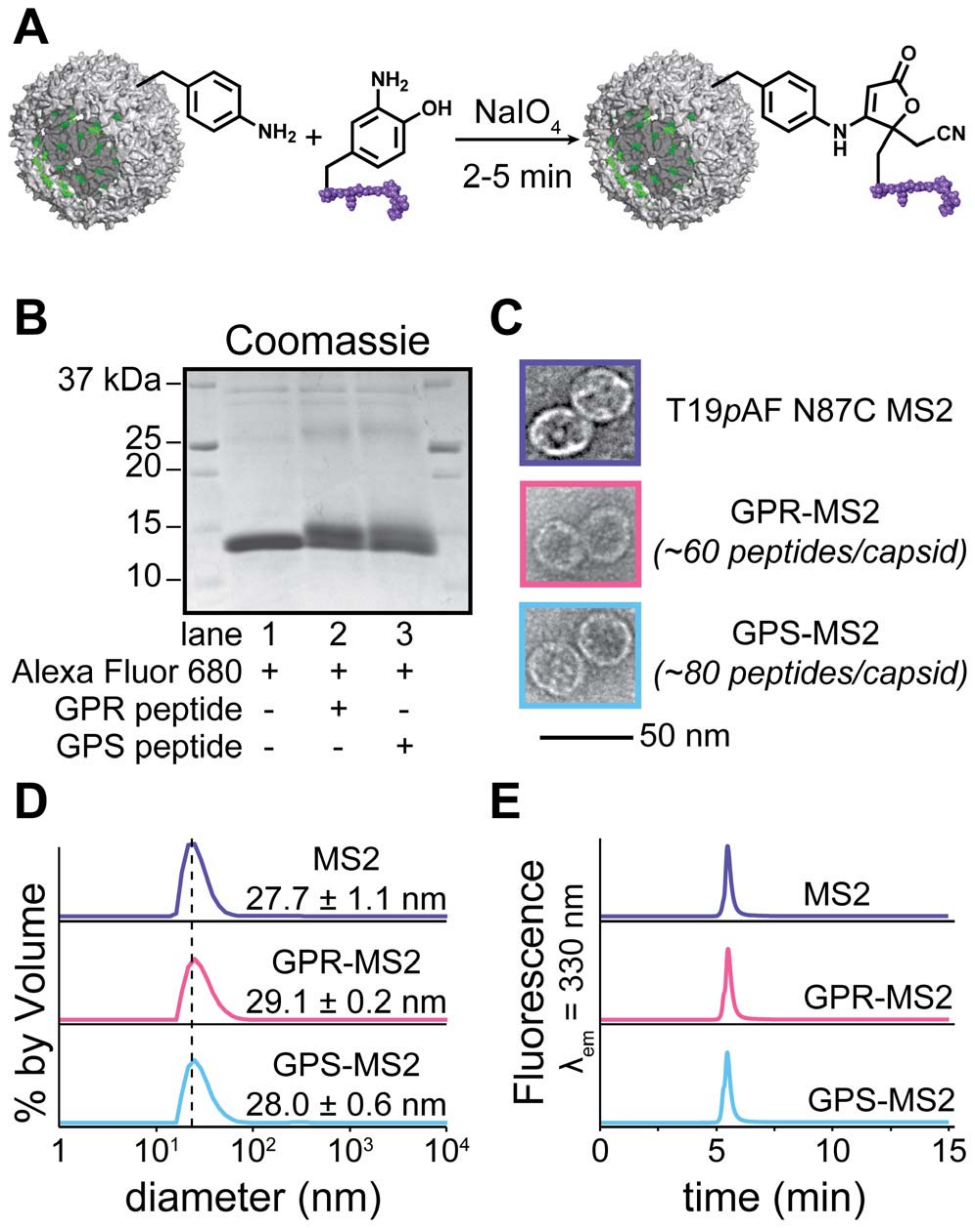
Characterization of MS2 conjugates. (a) The periodate-mediated oxidative coupling reaction takes place between *o*-aminophenol peptides and aniline containing MS2 capsids. (b) Alexa Fluor 680 and peptide-MS2 conjugates were analyzed by SDS-PAGE, with visualization of fluorescent (Figure S5) and Coomassie-stained bands (shown). Lanes 1–3 show disassembled MS2 monomers labeled with Alexa Fluor 680. In lane 2, the GPR peptide was added and in lane 3 the GPS peptide was added. The upper bands represent the fraction of the MS2 monomers conjugated to the peptides. (c) Transmission electron microscopy, (d) dynamic light scattering, and (e) size-exclusion chromatography (fluorescence: λ_ex_ = 280 nm, λ_em_ = 330 nm) of MS2, GPR-MS2, and GPS-MS2 confirmed that the capsids remained intact after modification. Wide-field TEM images appear in Figure S7.

To attach the peptide targeting groups to the MS2 capsids, we used a previously described double mutant of the capsid protein, Thr19*p*AF/Asn87Cys (T19*p*AF/N87C) and a recently reported NaIO_4_-mediated oxidative coupling of anilines and *o*-aminophenols (Figure 1a) [24], [36]. The aniline functionality was introduced onto the exterior surface of the capsids using the amber stop codon suppression method developed by the Schultz group [49], [50]. The *o*-aminophenol functionality was then incorporated into the peptide targeting group by chemically modifying a tyrosine residue after synthesis on the solid phase using standard Fmoc chemistry [48]. Removal of the side-chain protecting groups, azo coupling, and subsequent reduction with Na_2_S_2_O_4_ afforded the desired *o*-aminophenol-containing peptides (Figure 1c). The peptide modification was confirmed by mass spectrometry (MS), and the site of modification was verified through MS/MS analysis (Figures S1, S2, S3). The ability of the modified peptides to participate in the periodate-mediated oxidative coupling was confirmed by reacting the *o*-aminophenol-containing peptides with 1 equiv of *p*-toluidine and 10 equiv of NaIO_4_, followed by MS characterization (Figure S4). The *o*-aminophenol containing peptides were then coupled to aniline-containing MS2 capsids using the periodate-mediated oxidative coupling shown in Figure 2a. This bioconjugation strategy was chosen because it is fast, chemoselective and has been used successfully on complex biomolecular assemblies. By altering the reaction time from 30 s to 10 min and the number of peptide equivalents from 1 to 40, the extent of peptide labeling could be varied from 18 to 150 peptides/capsid (Figure S5a,b). The periodate-mediated coupling was optimized to install approximately 90 peptides on the surface of each capsid in 5 min, corresponding to ∼50% modification of MS2 monomers as determined using optical densitometry of SDS-PAGE gels (Figure 2b, Figure S6). This level of modification was achieved using 10 equiv of the *o*-aminophenol peptide and 100 equiv of NaIO_4_. No coupling was observed for control reactions with capsids lacking the *p*AF groups (both wt-MS2 and T19Y MS2), confirming the chemoselectivity of the method (Figure S5c). The coupling reactions were terminated by removal of the NaIO_4_ via gel filtration through Nap 5 Sephadex columns. After modification, the assembly state of the capsids was confirmed by transmission electron microscopy (TEM), dynamic light scattering (DLS), and size-exclusion chromatography (SEC, Figure 2c-e, Figure S7). Unmodified MS2 capsids were measured to have a diameter of 27.7±1.1 nm using DLS, while the modified capsids were determined to have diameters of 29.1±0.2 nm and 28.0±0.6 nm for GPR-MS2 and GPS-MS2, respectively. DLS also confirmed that the capsids had a normal size distribution [24], [51].

**Figure 3:**
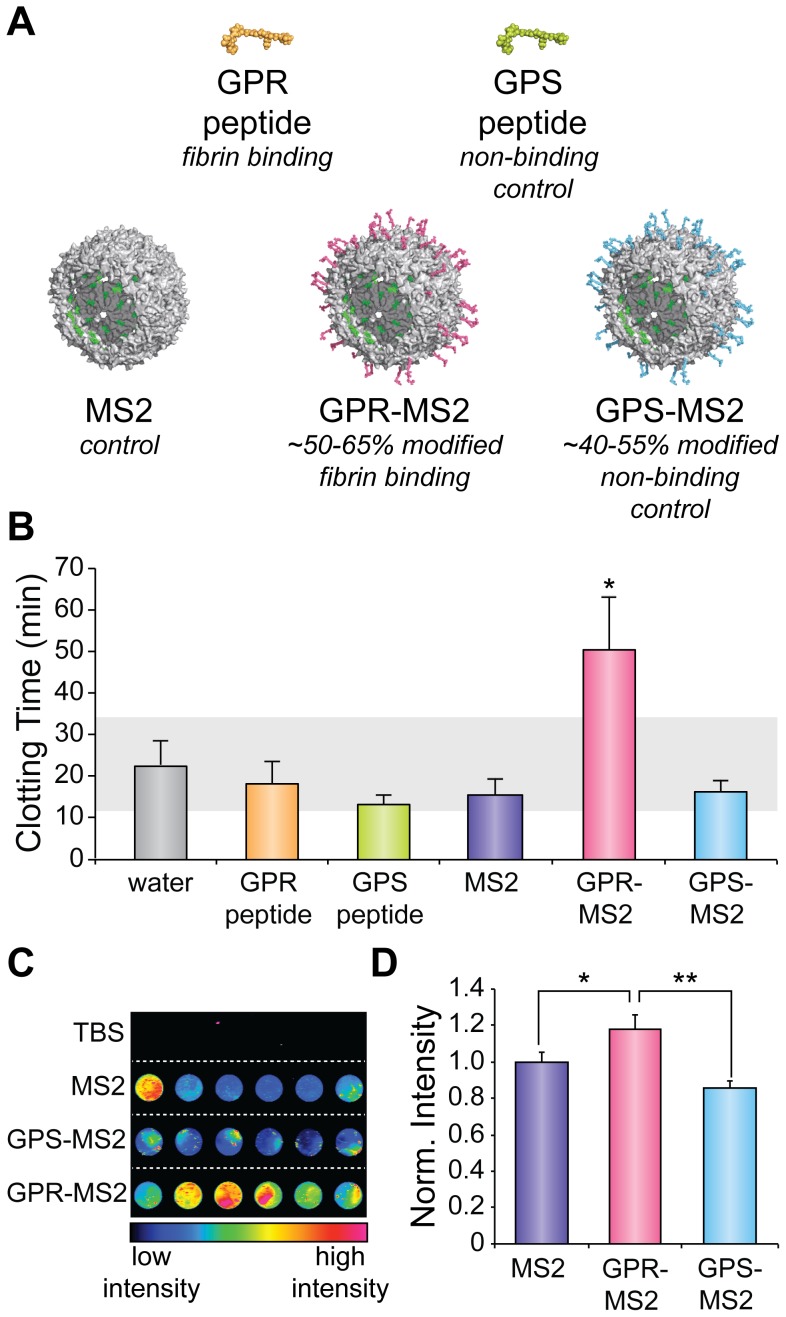
Fibrin targeting with GPR-MS2 conjugates. (a) Five different agents were tested for fibrin binding properties. (b) Binding to fibrin monomers was confirmed with a clotting inhibition assay. Clotting times are plotted as the average of several trials (n = 4-6), shown with the standard error. The grey bar indicates the 95% confidence interval for the water control. At a concentration of 20 µM peptide, (222 nM MS2 capsid), only GPR-MS2 increased the thrombin induced clotting time of fibrin (Welch's t-test, p<0.05). (c) A representative near-infrared fluorescence image of fibrin clots in a 96-well plate indicated the *in vitro* binding of agents to the clots. (d) Binding was quantified, showing that the differences in relative spot intensity (normalized to the MS2 capsid intensity) between the agents were statistically significant (*p<0.05; **p<0.01). Normalized intensities are shown with the standard error.

The ability of the peptides to bind to fibrin, both before and after conjugation to MS2, was first verified through the use of a clotting inhibition assay (Figure 3a). Agents capable of binding to fibrin were expected to slow thrombin-mediated clotting because the GPR peptide competitively binds to fibrin at the site necessary for initial fibrin aggregation. Fibrin clotting times in the presence of each of the agents were determined by measuring the extent of light scattering at 350 nm [52]. In agreement with previously published reports, free GPR was found to inhibit thrombin induced clotting at high concentrations (≥300 µM), but free GPS did not do so at any of the concentrations assayed (Figure S8) [38], [42]. Unmodified MS2 capsids and capsids labeled with ∼50% peptide (GPR-MS2 and GPS-MS2) were also assayed for their ability to inhibit clotting. At 222 nM MS2 capsid (40 µM in MS2 monomer and ∼20 µM in peptide), GPR-MS2 slowed aggregation, while none of the other agents showed an effect (Figure 3b). Clotting times more than doubled when treated with GPR-MS2 (51±29 min), whereas they remained unchanged when treated with free GPR (18±13 min), free GPS (13±6 min), MS2 (16±9 min) or GPS-MS2 (16±6 min). GPR-MS2 slowed fibrin polymerization at a peptide concentration approximately ten-fold less than that observed for the free peptide. We found that the multivalent display of the binding moiety resulted in increased avidity of the targeting peptide. While others have observed varying effects with a multivalent display of similar peptides (GPRP), perhaps both the spacing between the peptides and the size of the multivalent object play a role in the interaction of fibrin/fibrinogen with these peptides in multivalent displays [53], [54]. Others have observed similar increases in avidity with different multivalent objects [25], [55], [56], [57], [58].

The potential for MS2 VLPs to image fibrous clots was also tested *in vitro*. The introduction of a reactive cysteine to the interior surface of MS2 allowed for the conjugation of maleimide functionalized imaging agents. Previous work has shown that the capsids can be labeled with fluorescent dyes for optical imaging, Gd^3+^ and ^129^Xe for MRI, and ^64^Cu for PET using this strategy [24], [59], [60], [61]. For this work, MS2 capsids were modified with ∼60 copies of Alexa Fluor 680 to create near-infrared optical imaging agents. GPR or GPS peptides were then installed on the exterior surface using the oxidative coupling strategy described above [36]. This resulted in ∼45–55% labeling for GPR-MS2 (∼100 peptides/capsid) and GPS-MS2 (∼80 peptides/capsid).

To prepare well-defined clots for use in the binding assays, purified bovine fibrinogen was added to a series of Eppendorf tubes. Thrombin and CaCl_2_ were added to induce aggregation. After 1 h, the MS2-based agents were added to the fibrin clots at a capsid concentration of 111 nM (20 µM in MS2 monomer, approximately 10 µM in peptide). After 3 h of exposure at room temperature, the clots were rinsed three times with TBS and then transferred to a 96-well plate. The samples were imaged with an optical scanner using the 700 nm channel (Figure 3c). Clots treated with the fibrin-targeted MS2 capsids (GPR-MS2) showed greater signal than clots incubated with control agents (GPS-MS2 and MS2, Figure 3d). The signal intensity was normalized to the unmodified MS2 capsids to allow for comparison between experiments. While the MS2 capsids showed some background sticking, conjugation of the non-binding peptide to the exterior (GPS-MS2) greatly diminished the binding of the capsids. When the fibrin binding peptide was displayed on the exterior of MS2 (GPR-MS2), however, the capsids showed some enhanced binding relative to both the unmodified (MS2) and non-binding (GPS-MS2) capsids. The binding of the targeted capsid (GPR-MS2) was also evaluated in serum to mimic the complex milieu found *in vivo*. The fibrin binding of the targeted capsid was not inhibited by the addition of serum (Figure S9). The ability to bind at such low concentrations parallels results using single-chain antibody fragments, antibodies and other peptides [45], [46], [47], [62], [63]. However, in this case, significant amounts of imaging cargo can be simultaneously delivered for improved signal-to-noise ratios.

The free peptides were also labeled with Alexa Fluor 680 and tested for comparison. Before use, the modified peptides were HPLC purified and characterized by MS and MS/MS analysis (Figures S10, S11, S12, S13). The binding of the GPR and GPS peptides was compared to that of the Alexa Fluor 680 dye at concentrations ranging from 0.1 to 10 µM (Figure S14). At all concentrations tested, the peptides and dye non-specifically bound the fibrin clots. Additionally, the quantified signal-to-background ratio revealed that the free dye associated with the clots as well as, or better than, the free peptides. The binding of the free peptides was expected to be drastically perturbed by the Alexa Fluor 680 dye because the unlabeled peptides and dye have similar molecular weights. This did not affect the MS2-based agents, however, because the optical imaging agent was sequestered inside the capsid where it could not interact with fibrin.

### Conclusion

In this work, we describe the synthesis of a new VLP for the targeted imaging of thrombi. This study highlights the use of multivalent scaffolds to achieve increased binding and the use of an efficient oxidative coupling strategy for the installation of many copies of a given functional group in a short period of time. The MS2-based delivery vehicle was shown to bind fibrin, a key molecular target in blood clots, with greater efficiency than that of free peptides evaluated at the same concentration. The multivalent display of the peptide on the MS2 capsid improved the ability of the GPR peptide to inhibit thrombin-mediated clotting. The attachment of near-infrared dyes allowed for *in vitro* optical detection of fibrin binding. The modular synthesis of these agents allows for the facile modification of GPR-MS2 with MRI, PET, or optical imaging probes. The attachment of these other imaging agents will allow for the evaluation of these agents in future *in vivo* imaging studies.

## Materials and Methods

### General procedures and materials

Unless otherwise noted, the chemicals and solvents used were of analytical grade and were used as received from commercial sources. Water (dd-H_2_O) used as reaction solvent and in biological procedures was deionized using a Barnstead NANOpure purification system (ThermoFisher, Waltham, MA).

### Mass Spectrometry

Matrix assisted laser desorption-ionization time-of-flight mass spectrometry (MALDI-TOF MS) was performed on a Voyager-DE system (PerSeptive Biosystems, USA) and data were analyzed using Data Explorer software. Peptide samples were co-crystallized with α-cyano-4-hydroxycinnamic acid in 1∶1 acetonitrile (MeCN) to H_2_O with 0.1% trifluoroacetic acid (TFA). Electrospray ionization mass spectrometry (ESI-MS) of peptides was performed using an Agilent 1100 series LC pump outfitted with either an Agilent 6224 Time-of-Flight (TOF) LC/MS system or an API 150EX system (Applied Biosystems, USA) equipped with a Turbospray ion source. LC-ESI-MS of proteins and tandem mass spectrometry (MS/MS) of peptides were obtained from the UC Berkeley QB3/Chemistry Mass Spectrometry Facility. Protein bioconjugates were analyzed using an Agilent 1200 series liquid chromatograph (Agilent Technologies, USA) that was connected in-line with an LTQ Orbitrap XL hybrid mass spectrometer equipped with an Ion Max electrospray ionization source (ESI; Thermo Fisher Scientific, Waltham, MA). MS/MS analysis was accomplished with a using a Waters nanoAcquity ultra performance liquid chromatograph (UPLC)/quadrupole time-of-flight (Q-TOF) Premier instrument.

### High Performance Liquid Chromatography

HPLC was performed on Agilent 1100 Series HPLC Systems (Agilent Technologies, USA). Sample analysis for all HPLC experiments was achieved with an inline diode array detector (DAD) and inline fluorescence detector (FLD). Analytical and preparative reverse-phase HPLC of peptides and proteins was accomplished using a C18 stationary phase and a MeCN/H_2_O with 0.1% TFA gradient mobile phase. Analytical size exclusion chromatography of peptides and proteins was accomplished using a BioSep 4000S stationary phase with isocratic flow of aqueous buffer (10 mM phosphate buffer, pH 7.2).

### Gel Analyses

For protein analysis, sodium dodecyl sulfate-polyacrylamide gel electrophoresis (SDS-PAGE) was carried out on a Mini-Protean apparatus (Bio-Rad, Hercules, CA), using a 10–20% precast linear gradient polyacrylamide gel (Bio-Rad). The sample and electrode buffers were prepared according to Laemmli [64]. All protein electrophoresis samples were heated for 5–10 min at 95°C in the presence of 1,4-dithiothreitol (DTT) to ensure reduction of disulfide bonds. Gels were run for 75–90 minutes at 120 V to separate the bands. Commercially available markers (Bio-Rad) were applied to at least one lane of each gel for assignment of apparent molecular masses. Visualization of protein bands was accomplished by staining with Coomassie Brilliant Blue R-250 (Bio-Rad). For fluorescent protein conjugates, visualization was accomplished on a Typhoon 9410 variable mode imager (Amersham Biosciences) courtesy of Prof. Carolyn Bertozzi prior to gel staining. Coomasie stained protein gels were imaged on an EpiChem3 Darkroom system (UVP, USA).

### Dynamic Light Scattering

DLS measurements were obtained using a Malvern Instruments Zetasizer Nano ZS, courtesy of Prof. Jean Fréchet. Data plots were calculated from an average of three measurements, each of which consisted of 12 runs of 45 s each. Measurement data are presented as a volume plot, which weights larger dimensions by a factor of 10^3^ more than smaller dimensions. Samples were measured in 10 mM phosphate buffer, pH 7.2.

### Transmission Electron Microscopy

TEM images were obtained at the UC Berkeley Electron Microscope Lab (www.em-lab.berkeley.edu) using a FEI Tecnai 12 transmission electron microscope with 120 kV accelerating voltage. Protein samples were prepared for TEM analysis by pipetting 5 µL of the samples onto Formvar-coated copper mesh grids (400 mesh, Ted Pella, Redding, CA), after 3 min of equilibration the samples were then wicked with filter paper. The samples were then rinsed with dd-H_2_O. Subsequently, the grids were exposed to 8 µL of a 1% (w/v) aqueous solution of uranyl acetate for 90 s as a negative stain. After excess stain was removed, the grids were allowed to dry in air.

### General procedure for solid-phase peptide synthesis

Peptides were synthesized using standard Fmoc-based chemistry on Tentagel S-OH resin (Advanced ChemTech, Louisville, KY). The side chain protecting groups used were: Arg(Pbf), Cys(Trt), Lys(Boc), Ser(tBu), Tyr(tBu). The C-terminal amino acid (10 equiv) was preactivated at 0°C with 5 equivalents of diisopropylcarbodiimide (DIC) and then coupled to the resin with 0.1 equivalents of *N*,*N*-dimethylaminopyridine (DMAP) as a catalyst. Deprotection of the Fmoc groups was performed with a 20 min incubation in a 20% v/v piperidine in dimethylformamide (DMF) solution. Coupling reactions were carried out using 20 equivalents of amino acid with 10 equivalents of 2-(6-chloro-1-*H*-benzotriazole-1-yl)-1,1,3,3-tetramethylaminium hexafluorophosphate (HCTU) [48] and 20 equivalents of *N*,*N*-diisopropylethylamine (DIPEA) in DMF for 20 min. Side-chain deprotection was accomplished using a 1–2 h incubation with either a 95∶2.5∶2.5 ratio of TFA to H_2_O to triisopropylsilane (TIPS) (peptides without cysteine) or a 90∶5∶2.5∶2.5 ratio of TFA to phenol to H_2_O to TIPS (peptides containing cysteine). Peptides were cleaved from the resin by a 30–45 min incubation with a 100 mM sodium hydroxide solution. The resulting basic solution was neutralized with 100 mM phosphate buffer, pH 6.5.

### General procedure for azo coupling to peptides

To a portion of resin-bound peptide (appx. 20 mg) in 900 µL of 100 mM phosphate buffer, pH 9.0 was added 100 µL of a saturated solution of 4-nitrobenzenediazonium tetrafluoroborate (300 mM, Sigma-Aldrich) in MeCN at 4°C. After rotation for 30 min at 4°C, the resin was rinsed with MeCN and DMF until no color remained in solution. The peptides were then cleaved from the resin, and the modification was confirmed by MALDI-TOF MS (Figures S1–S2).

### General procedure for dithionite reduction of azo peptides

To a solution of azo-modified peptide (appx. 1 mM) in 100 mM phosphate buffer, pH 6.5 was added an equal volume of a freshly prepared solution of sodium dithionite (115 mM) in 100 mM phosphate buffer, pH 7.2. After 10 min, the solution was applied to a C18 Sep-Pak pre-conditioned with methanol then 0.1% aqueous TFA. After washing with aqueous 0.1% TFA and 5% MeCN in aqueous 0.1% TFA, the peptide was eluted with MeCN. After removing the MeCN under reduced pressure the solid peptide was dissolved in 100 µL of 10 mM phosphate buffer, pH 6.5. The *o*-aminophenol peptide was characterized by MALDI-TOF or LC-ESI MS (Figures S1–S2) and stored at 4°C. The site of modification was confirmed for the GPR peptide by MS/MS analysis (Figure S3).

### General procedure for maleimide modification of peptides

To a 1.75 mM solution of cleaved peptide in 10 mM phosphate buffer, pH 8.0 was added 1.1 equivalents of *tris*(2-carboxyethyl)phosphine hydrochloride (0.5 M solution, pH 7.0). After 15 min, 0.6 equivalents of maleimide (Alexa Fluor 680 C_2_-maleimide, 20 mM in DMSO) was added and the solution was briefly vortexed. The peptides were incubated in the dark at room temperature overnight. The peptides were then purified by HPLC using a C18 Gemini column (5 micron, 250×7.8 mm, Phenomenex, Torrance, CA). The purified peptides were characterized by LC-ESI-MS and MS/MS (Figures S9–12).

### Expression and Purification of T19*p*AF N87C MS2

The protein was expressed and purified following a previously published protocol [34]. After purification, approximately 10 mg of protein were obtained per L of culture.

### General procedure for oxidative coupling of *o*-aminophenol peptides to pAF MS2

To a solution of T19*p*AF N87C MS2 (10 µM) in 10 mM phosphate buffer, pH 6.5 was added 5–10 equivalents of *o*-aminophenol peptide (50–100 µM). The solution was briefly vortexed and then 10 equivalents (relative to the *o*-aminophenol) of sodium periodate was added. After 5 min, the reaction was purified on a Nap 5 Sephadex size exclusion column (GE Healthcare) according to the manufacturer's instructions and eluted in 10 mM phosphate buffer, pH 7.2. Any remaining periodate and free peptide were removed using a 0.5 mL centrifugal filter with a molecular weight cut off (MWCO) of 100 kDa (Millipore). The samples were concentrated to 50 µL and then diluted 10-fold with 10 mM phosphate buffer, pH 7.2. This process was repeated 5–10 times depending on the concentration of peptide used. Modification was monitored by SDS-PAGE and quantified using optical densitometry (Figures S5–6).

### General procedure for dual modification of T19*p*AF N87C MS2

Dual modification always started with maleimide modification of the interior cysteine. A solution of T19*p*AF N87C MS2 (100 µM) in 10 mM phosphate buffer, pH 7.2 was incubated with 1 equivalent of Alexa Fluor 680 C_2_-maleimide (38 mM in DMSO) for 2 h. Unreacted dye was removed with a Nap 10 Sephadex size exclusion column (GE Healthcare) and by repeated centrifugal filtration against a 100 kDa MWCO membrane. Exterior modification with an *o*-aminophenol peptide was performed as described in the main text and as described above. Modification was monitored by SDS-PAGE (Figure 2).

### Inhibition of fibrin clot formation

Bovine fibrinogen (≥75% clottable, Sigma Aldrich, USA) was dissolved in 0.9% saline solution at 37°C at a concentration of 12 mg/mL. Bovine thrombin (100 U/mL) was dissolved in phosphate buffered saline (PBS) and added into wells of a 96-well polystyrene half area plate (Costar, Corning, NY) to a final concentration of 0.25 U/well. Assayed agents (H_2_O (control), GPR peptide, GPS peptide, MS2, GPR-MS2, and GPS-MS2) were added to the wells (72.5 µL/well, n = 4–6) to a final concentration of 20 µM peptide (40 µM in MS2 monomer for MS2, GPR-MS2, and GPS-MS2 for an effective peptide concentration of 20 µM and capsid concentration of 222 nM). Immediately preceding absorbance measurements, 25 µL of the fibrinogen solution was added to each well (0.3 µg/well). The absorbance at 350 nm was monitored once a minute for 75 min using a SpectraMax M3 microplate reader (Molecular Devices, Sunnyvale, CA), courtesy of Prof. Carolyn Bertozzi. Additional concentrations of peptide (10 µM to 1.7 mM) were evaluated for their ability to inhibit fibrin clot formation in a similar manner (Figure S8). The clotting time was determined to be the time at which the absorbance first approached the horizontal asymptote. Statistical significance was evaluated by a one-tailed Student's t-test with unequal variance (n = 4–6).

### Binding to fibrin clots

Bovine fibrinogen (≥75% clottable, Sigma Aldrich, USA) was dissolved in 50 mM Tris, pH 7.4, 150 mM sodium chloride (TBS) and dialyzed against TBS with 5 mM sodium citrate (TBS-citrate). The resulting fibrinogen solution was adjusted to 4 mg/mL by measuring the absorbance at 280 nm (a 1 mg/mL fibrinogen solution has an absorbance of 1.512 OD units) [65]. The fibrinogen solution (50 µL) was aliquoted into Eppendorf tubes. Subsequently, calcium chloride (10 µL of a 70 mM solution) and 0.4 U of bovine thrombin (10 U/mL in TBS) were added to each tube to initiate clotting. After incubation at room temperature for 1 h, 100 µL of Alexa Fluor 680 labeled agent (MS2, GPR-MS2 or GPS-MS2) was added to each clot (n = 3, with 4–6 replicates per experiment) for a final concentration of 20 µM MS2 monomer (111 nM MS2 capsid and approximately 10 µM peptide). The agents were allowed to bind for 3 h at room temperature and then the clots were washed 3 times with 200 µL TBS. The clots were transferred to a 96-well polystyrene plate (Costar, Corning, NY) and centrifuged at 4000 rpm for 10 min. The plate was imaged using an Odyssey CLx infrared imaging system (Li-Cor, Lincoln, NE) courtesy of Prof. Jay Groves. The binding of GPR-MS2 was also evaluated in fetal bovine serum (FBS) following the protocol outlined above (8 replicates). The resulting images were quantified using ImageJ 1.42q software (NIH) using the Microarray Profile plugin to select regions-of-interest. The image was pseudo-colored in ImageJ. Additionally, AlexaFluor 680 labeled peptide and free AlexaFluor 680 C_2_-maleimide (10, 1, and 0.1 µM) were tested for their binding to fibrin clots (Figure S14). Statistical significance was evaluated by a one-tailed Student's t-test with unequal variance (n = 3, with 4-6 replicates per experiment).

## Supporting Information

Figure S1 **Conversion of GPR peptide C-terminal tyrosine to an** ***o*** **-aminophenol.** MALDI-TOF MS was used to monitor the modification of (a) the unmodified peptide to (b) the azo-peptide with 4-nitrobenzenediazonium tetrafluoroborate, and then subsequent sodium dithionite reduction to (c) the *o*-aminophenol peptide.
(TIFF)

Figure S2 **Conversion of GPS peptide C-terminal tyrosine to an** ***o*** **-aminophenol.** MALDI-TOF MS was used to monitor the modification of (a) the unmodified peptide to (b) the azo-peptide with 4-nitrobenzenediazonium tetrafluoroborate, and then subsequent sodium dithionite reduction to (c) the *o*-aminophenol peptide.
(TIFF)

Figure S3 **MS/MS analysis of** ***o*** **-aminophenol modified GPR peptide.** (a) MS/MS of the GPR peptide and (b) fragments shown in blue are y ions (modified fragments), fragments shown in red are b ions (unmodified fragments), fragments shown in green are imminium ions (both modified and unmodified fragments), and fragments shown in purple are a ions (unmodified fragments). Products of neutral loss of molecules of water or ammonia are denoted by asterisks. The analysis is consistent with the modification of the C-terminal tyrosine.
(TIFF)

Figure S4 **Oxidative coupling to** ***o*** **-aminophenol containing peptides.** (a) The GPR peptide was coupled to 1 equiv of *p*-toluidine in 5 min in the presence of 10 equiv of NaIO_4_. The reaction was quenched with *tris*(2-carboxyethyl)phosphine hydrochloride (0.5 M solution, pH 7.0) and the mass of the coupled peptide was confirmed by ESI-MS. (b) The GPS peptide was similarly coupled to *p*-toluidine and the product was confirmed by ESI-MS.
(TIFF)

Figure S5 **Oxidative coupling optimization screen with GPR peptide.** (a) Coupling of varying concentrations of GPR peptide with 25 µM T19*p*AF N87C MS2 in the presence of 1 mM sodium periodate in 10 mM phosphate buffer, pH 7.2 for 10 min. Varying the peptide concentration allowed for control over the level of modification. The secondary modification is presumed to be due to a second addition of the *o*-aminophenol to the exterior aniline as this has occasionally been observed with small molecules. (b) A time course experiment with 25 µM T19*p*AF N87C MS2, 100 µM GPR peptide, and 1 mM sodium periodate in 10 mM phosphate buffer, pH 7.2. Each time point was quenched by the addition of 5 µL of loading buffer. The reaction reached maximum conversion after 2.5–5 min. (c) Negative controls confirm the specificity of the reaction for the aniline side-chain. Additionally, both coupling partners and periodate were necessary for modification to be observed.
(TIFF)

Figure S6 Alexa Fluor 680 and peptide-MS2 conjugates were analyzed by SDS-PAGE, with visualization of fluorescent and Coomassie-stained bands. Lanes 1-3 show disassembled MS2 monomers labeled with Alexa Fluor 680. In lane 2, the GPR peptide was added and in lane 3 the GPS peptide was added. The upper bands represent the fraction of the MS2 monomers conjugated to the peptides. (a) Shows the full gel image and (b) shows a zoomed in view of the region of interest. Unmarked lanes contain either recombinant prestained protein standards (Bio-Rad) or the negative controls seen in Figure S5c. The protein standards were visible under Coomassie staining conditions, but the standards were not fluorescent so they were not observed in the fluorescence image. (c) Another gel shows the shift observed for Alexa Fluor 680 labeled capsids as well as the additional shift observed after subsequent conjugation of peptides. Lane 4 shows unmodified MS2 monomers and lane 5 shows Alexa Fluor 680 labeled monomers. The Alexa Fluor 680 labeled MS2 was subsequently modified with peptides, and lanes 6 and 7 show the addition of the GPR and GPS peptides, respectively.
(TIFF)

Figure S7 Wide-field TEM images of (a) T19*p*AF N87C MS2, (b) GPR-MS2 (∼33% modified, ∼60 peptides/capsid), and (c) GPS-MS2 (∼45% modified, ∼80 peptides/capsid) revealed that the capsids remained intact after the oxidative coupling of peptides to the exterior surface. The observed debris may be due to the age of the capsids or the TEM grids; images taken shortly after purification showed little debris.
(TIFF)

Figure S8 **Inhibition of thrombin induced fibrin clotting with varying concentrations of free GPR and GPS peptides.** Only at high concentrations (≥ ∼ 300 µM) of GPR peptide was an effect observed. The concentrations used in Figure 3b of the main text (10–60 µM, starred on graph) showed no effect for either peptide.
(TIFF)

Figure S9 The binding of GPR-MS2 (20 µM MS2 monomer, 111 nM MS2 capsid, and ∼10 µM peptide) in fetal bovine serum (FBS) was compared to binding in Tris buffered saline (TBS). The addition of serum did not inhibit binding of the targeted capsids.
(TIFF)

Figure S10 **Modification of GPR peptide C-terminal cysteine with Alexa Fluor 680 C_2_-maleimide.** ESI-MS confirmed the mass of (a) the unmodified peptide and showed the mass of (b) Alexa Fluor 680 modification of the GPR peptide.
(TIFF)

Figure S11 **Modification of GPS peptide C-terminal cysteine with Alexa Fluor 680 C_2_-maleimide.** ESI-MS confirmed the mass of (a) the unmodified peptide and showed the mass of (b) Alexa Fluor 680 modification of the GPS peptide.
(TIFF)

Figure S12 **MS/MS analysis of Alexa Fluor 680 modified peptide.** (a) MS/MS of the GPR peptide and (b) fragments shown in blue are y ions (modified fragments), fragments shown in red are b ions (unmodified fragments), fragments shown in green are imminium ions (both modified and unmodified fragments), and fragments shown in purple are a ions (unmodified fragments). The analysis is consistent with the modification of the C-terminal cysteine.
(TIFF)

Figure S13 **MS/MS analysis of Alexa Fluor 680 modified GPS peptide.** (a) MS/MS of the GPS peptide and (b) fragments shown in blue are y ions (modified fragments), fragments shown in red are b ions (unmodified fragments), fragments shown in green are imminium ions (both modified and unmodified fragments), and fragments shown in purple are a ions (unmodified fragments). The analysis is consistent with the modification of the C-terminal cysteine.
(TIFF)

Figure S14 Alexa Fluor 680 labeled peptides and Alexa Fluor 680 were assayed for their binding to fibrin clots. At all concentrations tested (10, 1, and 0.1 µM) the two peptides showed no difference in binding. Additionally, the free dye associated non-specifically to the clots as well as, or better than, the peptides modified with the fluorescent dye. The average signal-to-background (n = 6) is shown with the standard error.
(TIFF)
