# Supplementary figures and images for: Multivalent Viral Capsids with Internal Cargo for Fibrin Imaging
Source: PLoS One. 2014 Jun 24;9(6):e100678. doi: 10.1371/journal.pone.0100678 (PMC4069081; doi:10.1371/journal.pone.0100678)

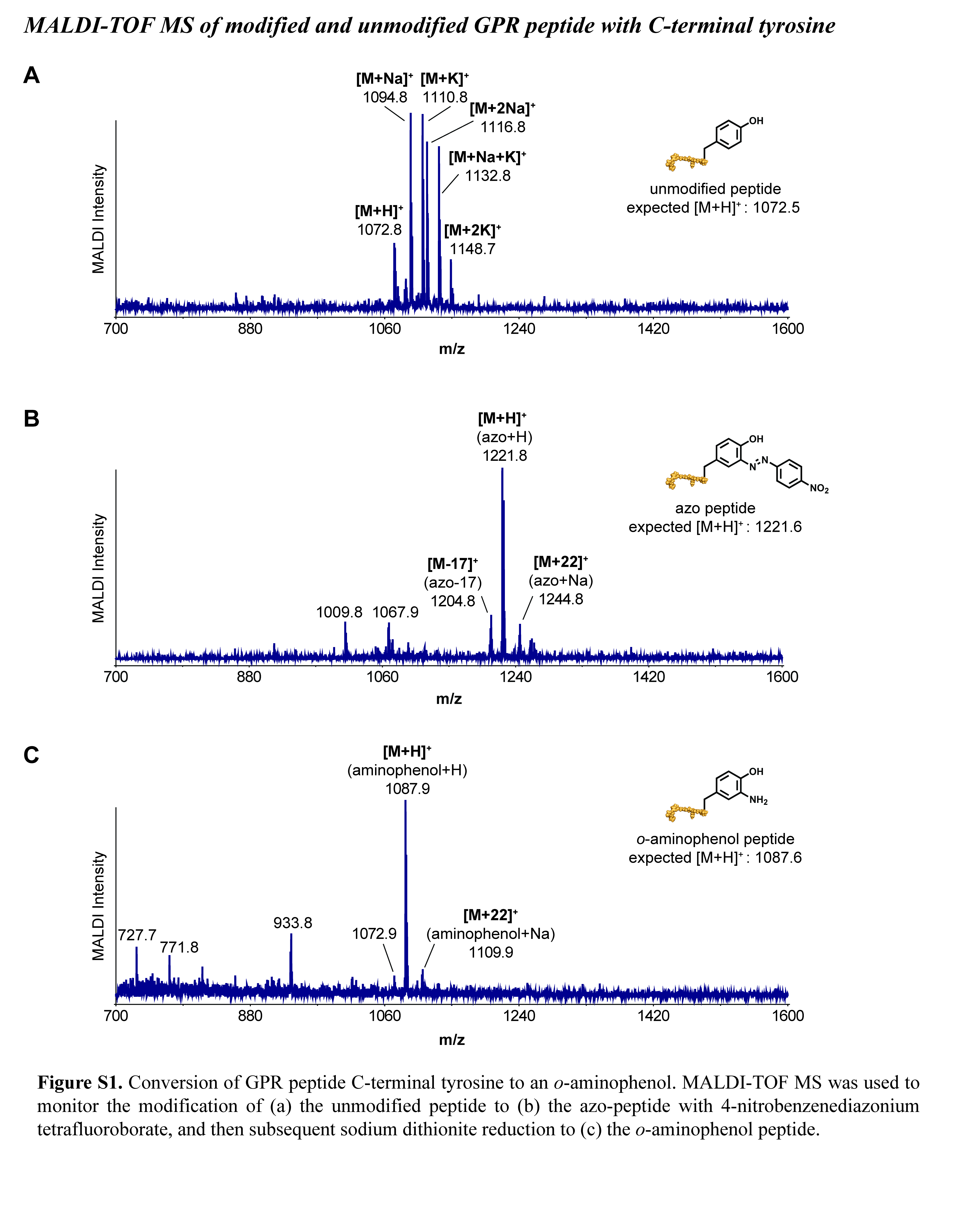

Supplement: Figure S1 — Conversion of GPR peptide C-terminal tyrosine to an o -aminophenol. MALDI-TOF MS was used to monitor the modification of (a) the unmodified peptide to (b) the azo-peptide with 4-nitrobenzenediazonium tetrafluoroborate, and then subsequent sodium dithionite reduction to (c) the o-aminophenol peptide. (TIFF) [file pone.0100678.s001.tiff]

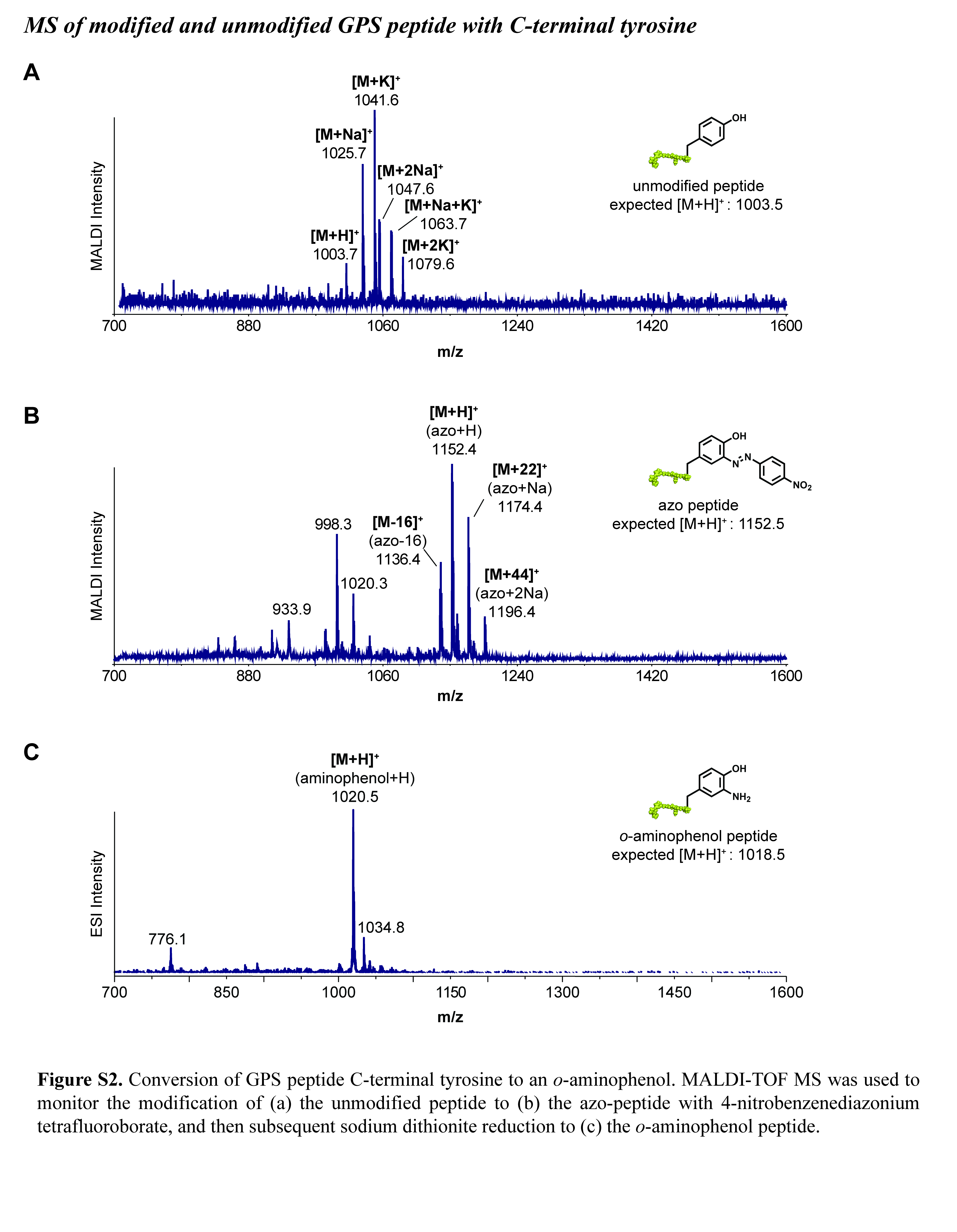

Supplement: Figure S2 — Conversion of GPS peptide C-terminal tyrosine to an o -aminophenol. MALDI-TOF MS was used to monitor the modification of (a) the unmodified peptide to (b) the azo-peptide with 4-nitrobenzenediazonium tetrafluoroborate, and then subsequent sodium dithionite reduction to (c) the o-aminophenol peptide. (TIFF) [file pone.0100678.s002.tiff]

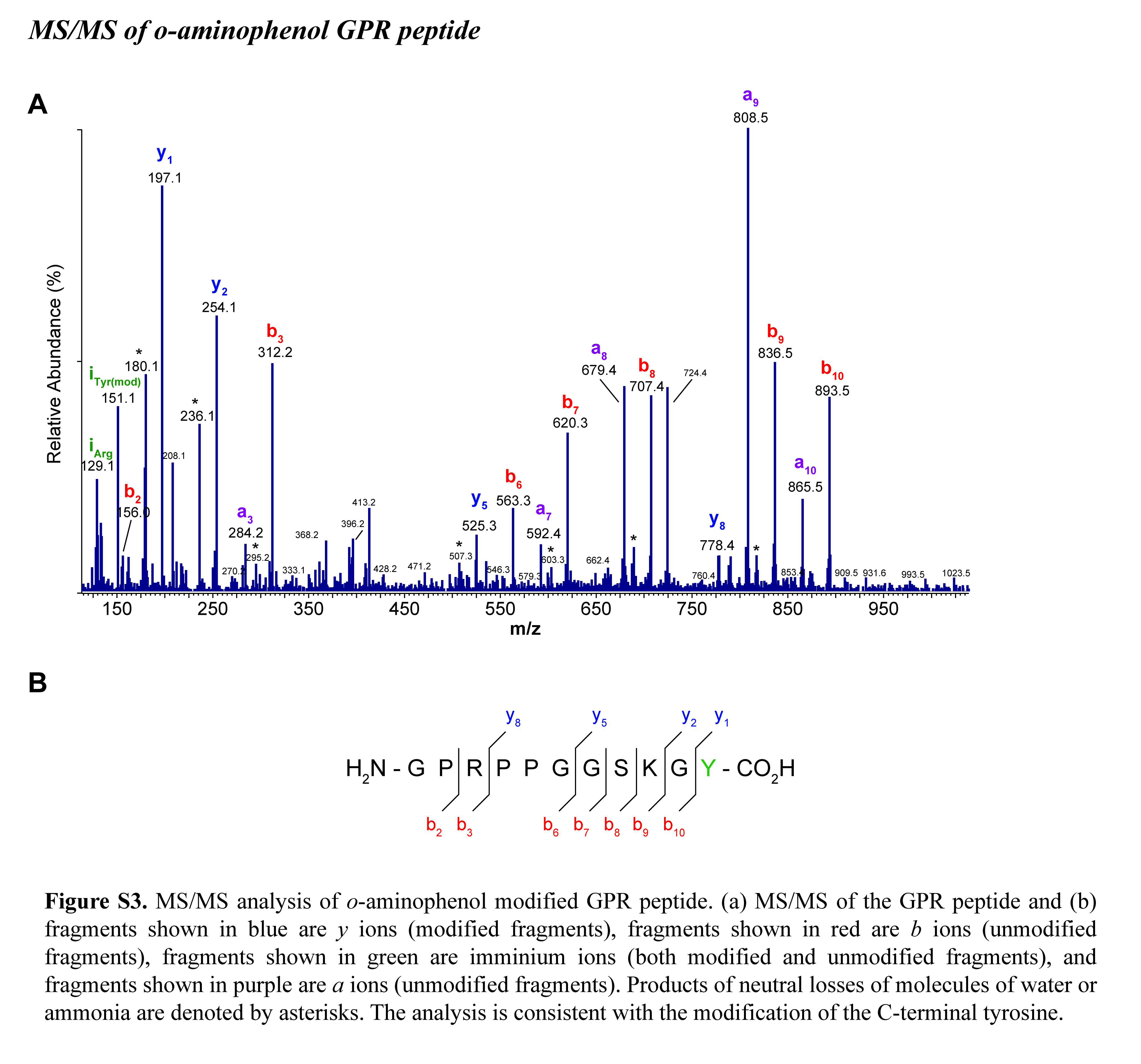

Supplement: Figure S3 — MS/MS analysis of o -aminophenol modified GPR peptide. (a) MS/MS of the GPR peptide and (b) fragments shown in blue are y ions (modified fragments), fragments shown in red are b ions (unmodified fragments), fragments shown in green are imminium ions (both modified and unmodified fragments), and fragments shown in purple are a ions (unmodified fragments). Products of neutral loss of molecules of water or ammonia are denoted by asterisks. The analysis is consistent with the modification of the C-terminal tyrosine. (TIFF) [file pone.0100678.s003.tiff]

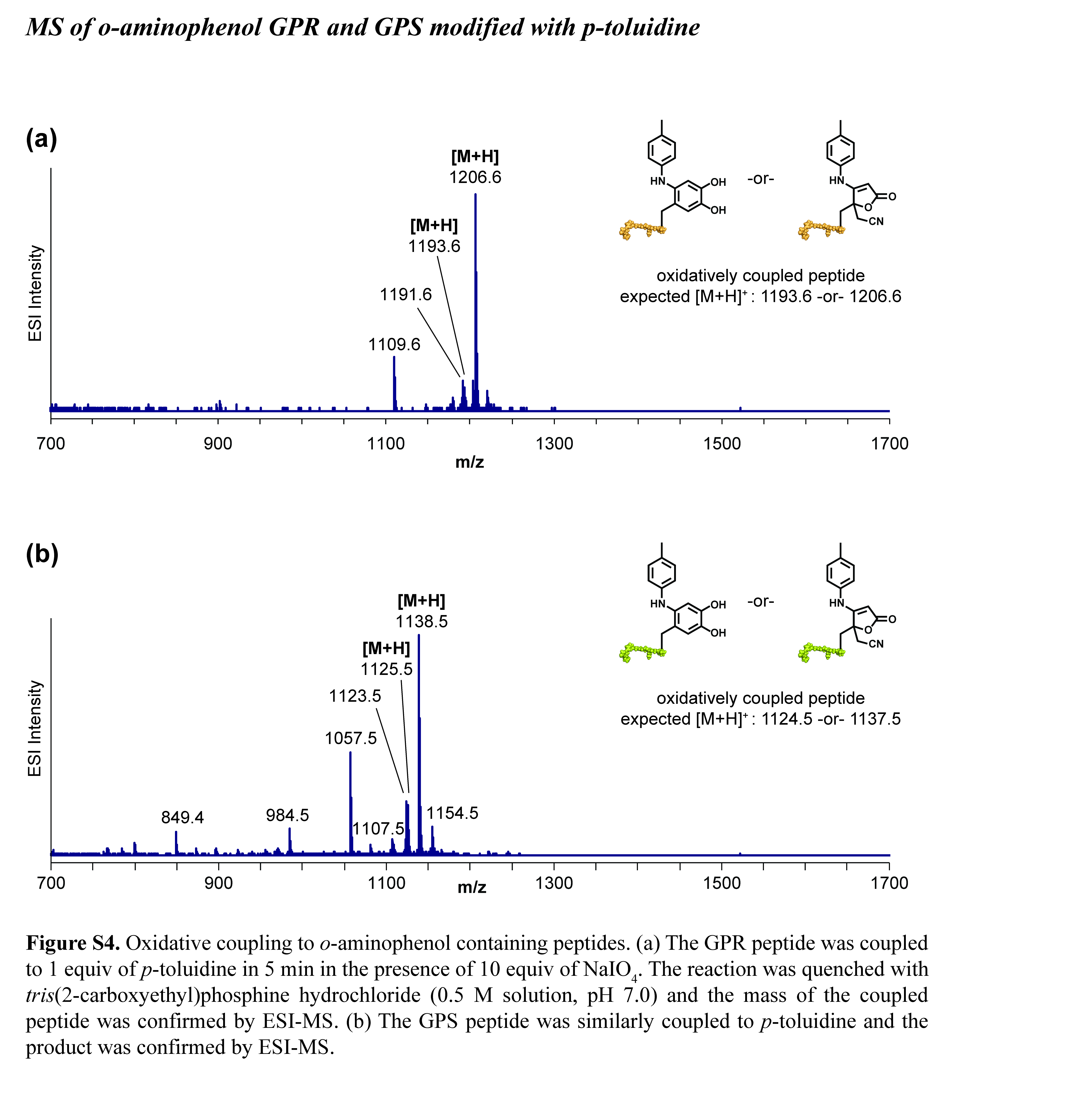

Supplement: Figure S4 — Oxidative coupling to o -aminophenol containing peptides. (a) The GPR peptide was coupled to 1 equiv of p-toluidine in 5 min in the presence of 10 equiv of NaIO4. The reaction was quenched with tris(2-carboxyethyl)phosphine hydrochloride (0.5 M solution, pH 7.0) and the mass of the coupled peptide was confirmed by ESI-MS. (b) The GPS peptide was similarly coupled to p-toluidine and the product was confirmed by ESI-MS. (TIFF) [file pone.0100678.s004.tiff]

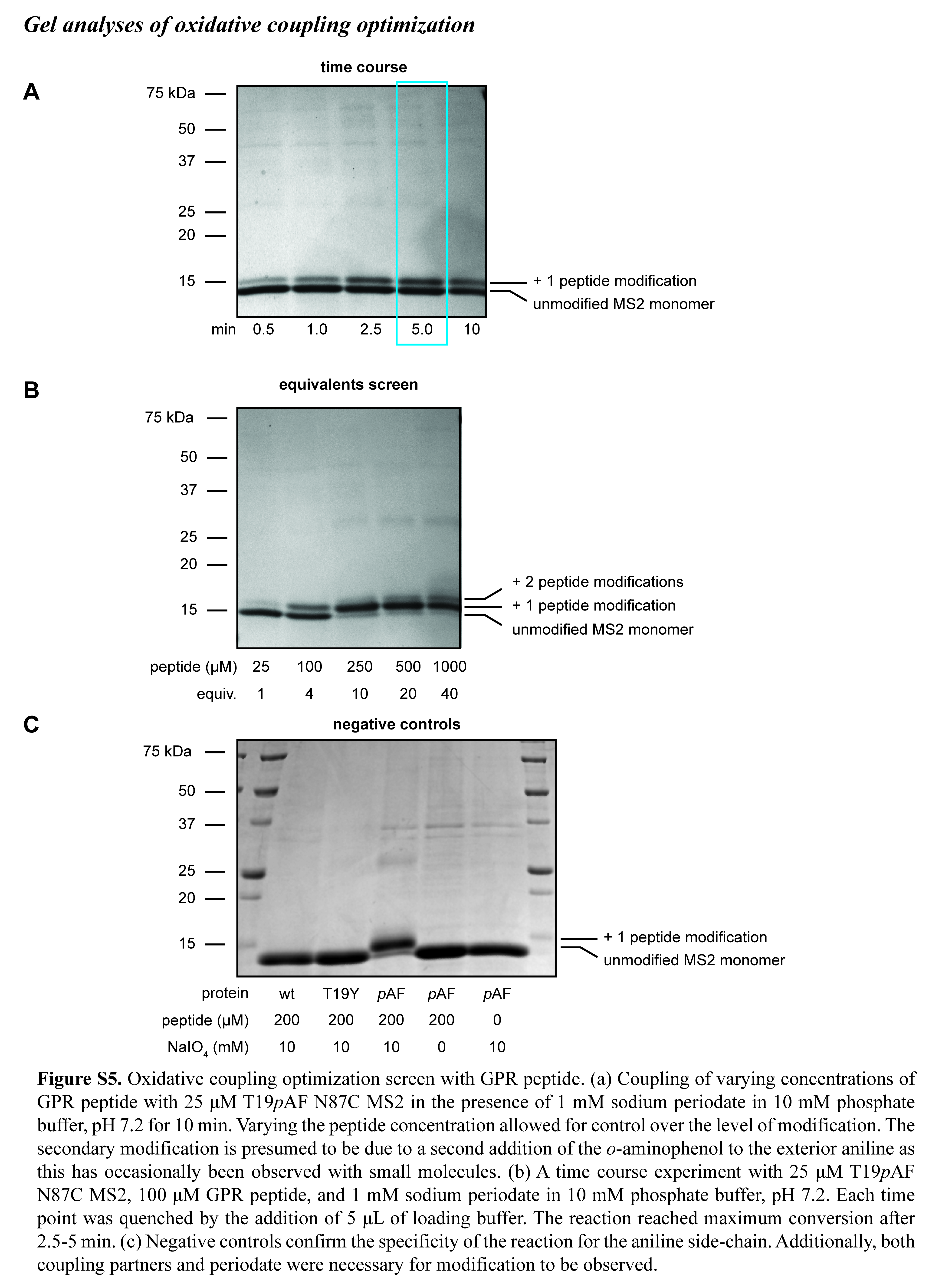

Supplement: Figure S5 — Oxidative coupling optimization screen with GPR peptide. (a) Coupling of varying concentrations of GPR peptide with 25 µM T19pAF N87C MS2 in the presence of 1 mM sodium periodate in 10 mM phosphate buffer, pH 7.2 for 10 min. Varying the peptide concentration allowed for control over the level of modification. The secondary modification is presumed to be due to a second addition of the o-aminophenol to the exterior aniline as this has occasionally been observed with small molecules. (b) A time course experiment with 25 µM T19pAF N87C MS2, 100 µM GPR peptide, and 1 mM sodium periodate in 10 mM phosphate buffer, pH 7.2. Each time point was quenched by the addition of 5 µL of loading buffer. The reaction reached maximum conversion after 2.5–5 min. (c) Negative controls confirm the specificity of the reaction for the aniline side-chain. Additionally, both coupling partners and periodate were necessary for modification to be observed. (TIFF) [file pone.0100678.s005.tiff]

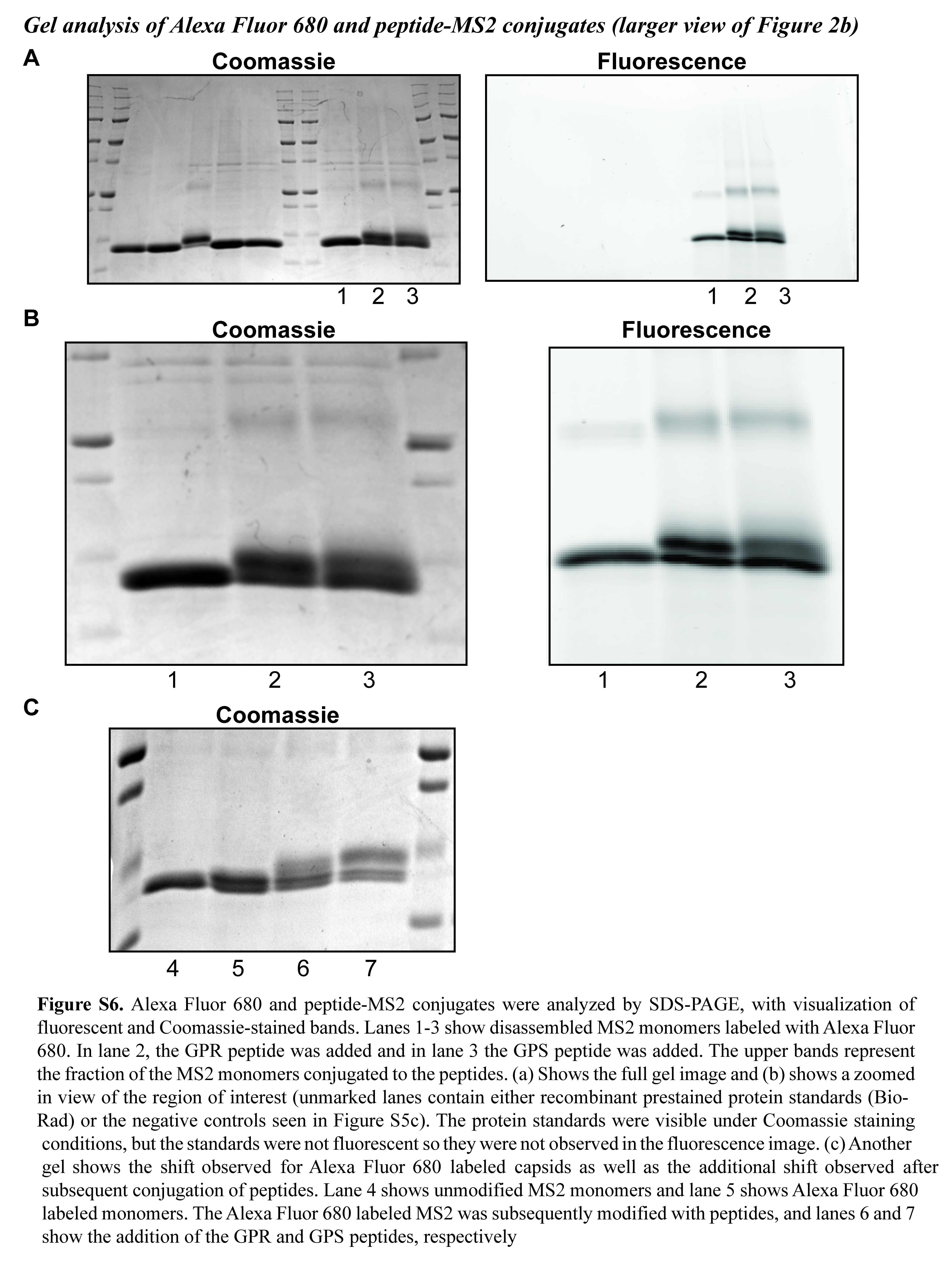

Supplement: Figure S6 — Alexa Fluor 680 and peptide-MS2 conjugates were analyzed by SDS-PAGE, with visualization of fluorescent and Coomassie-stained bands. Lanes 1-3 show disassembled MS2 monomers labeled with Alexa Fluor 680. In lane 2, the GPR peptide was added and in lane 3 the GPS peptide was added. The upper bands represent the fraction of the MS2 monomers conjugated to the peptides. (a) Shows the full gel image and (b) shows a zoomed in view of the region of interest. Unmarked lanes contain either recombinant prestained protein standards (Bio-Rad) or the negative controls seen in Figure S5c. The protein standards were visible under Coomassie staining conditions, but the standards were not fluorescent so they were not observed in the fluorescence image. (c) Another gel shows the shift observed for Alexa Fluor 680 labeled capsids as well as the additional shift observed after subsequent conjugation of peptides. Lane 4 shows unmodified MS2 monomers and lane 5 shows Alexa Fluor 680 labeled monomers. The Alexa Fluor 680 labeled MS2 was subsequently modified with peptides, and lanes 6 and 7 show the addition of the GPR and GPS peptides, respectively. (TIFF) [file pone.0100678.s006.tiff]

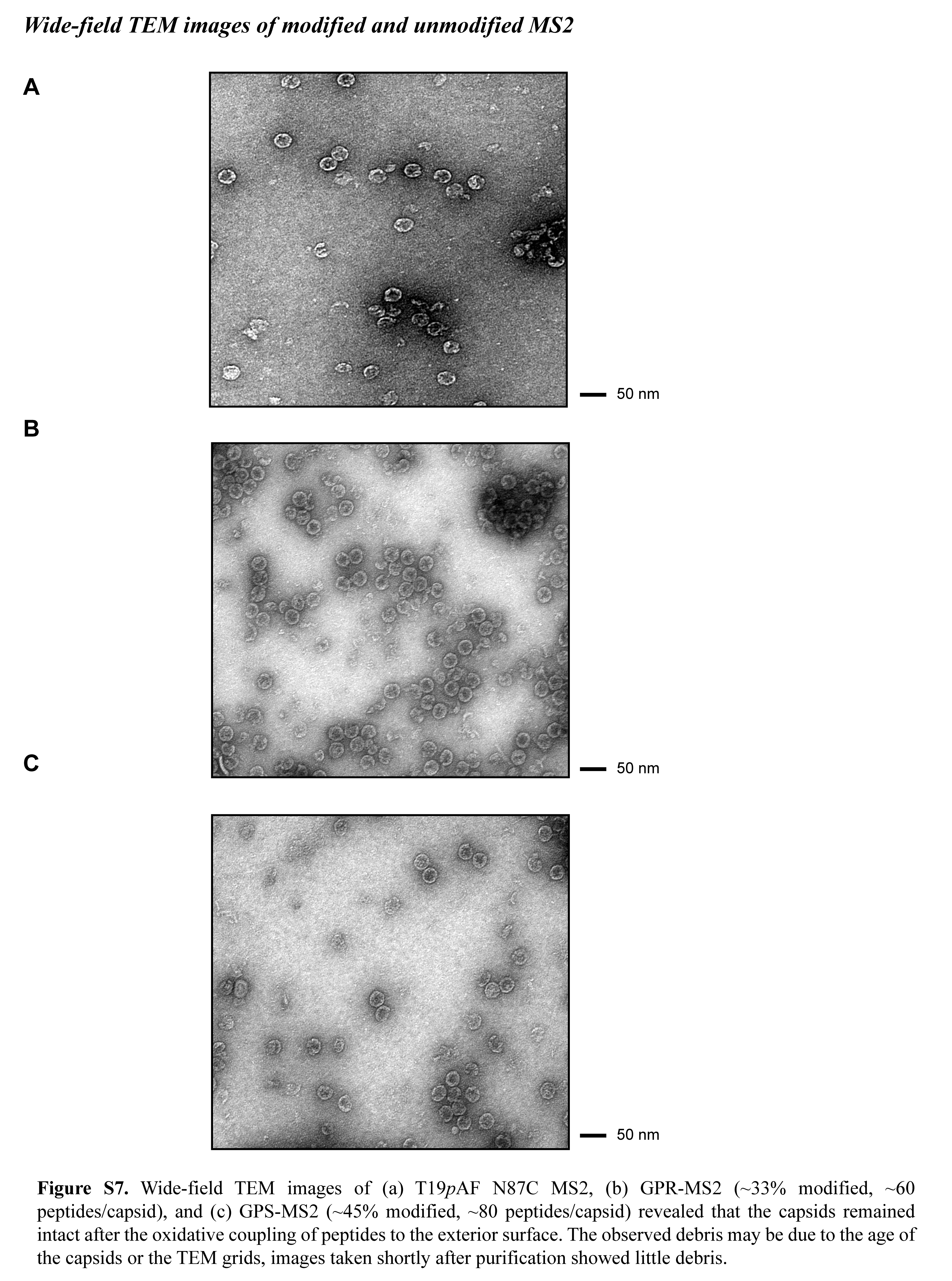

Supplement: Figure S7 — Wide-field TEM images of (a) T19pAF N87C MS2, (b) GPR-MS2 (∼33% modified, ∼60 peptides/capsid), and (c) GPS-MS2 (∼45% modified, ∼80 peptides/capsid) revealed that the capsids remained intact after the oxidative coupling of peptides to the exterior surface. The observed debris may be due to the age of the capsids or the TEM grids; images taken shortly after purification showed little debris. (TIFF) [file pone.0100678.s007.tiff]

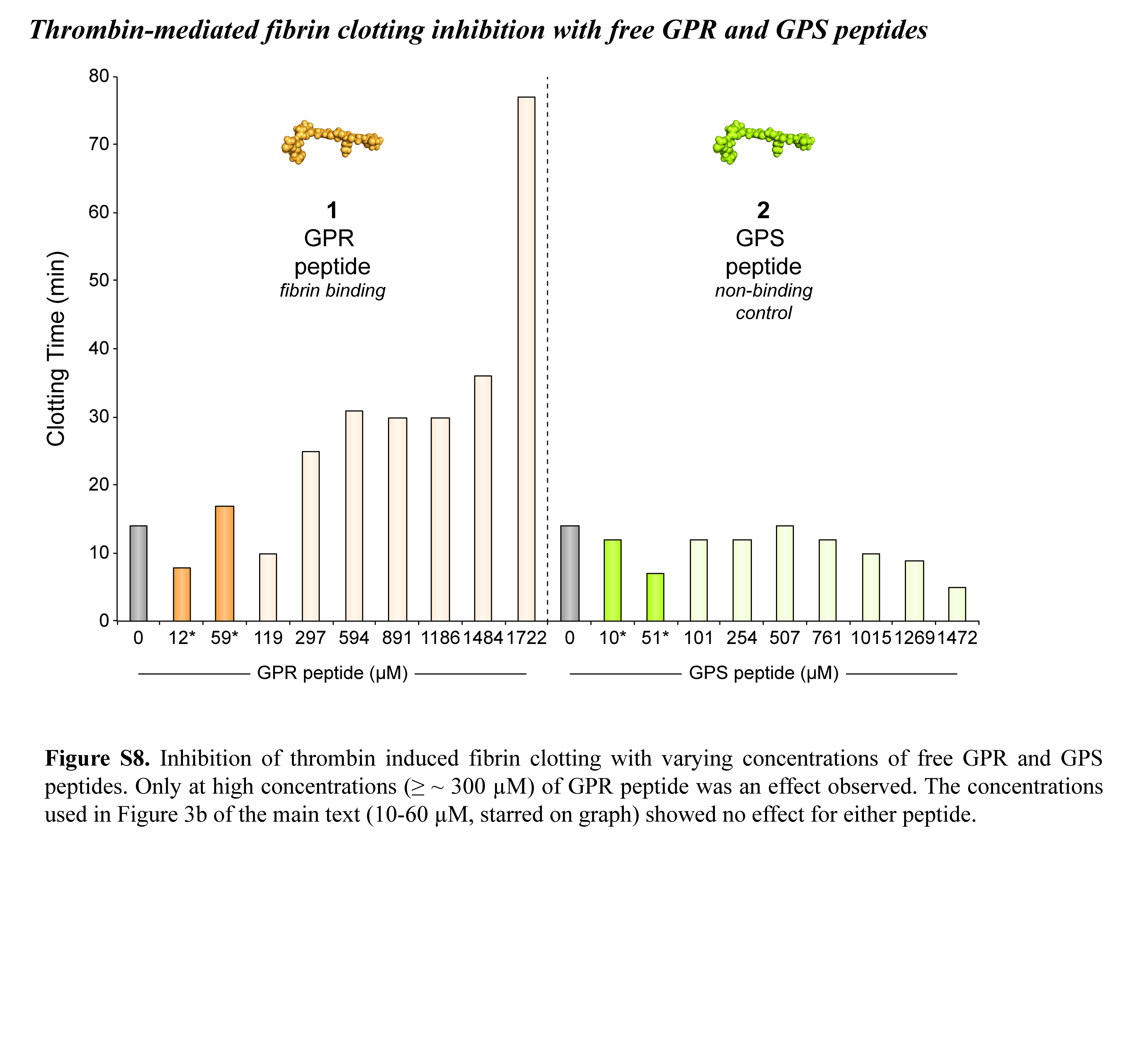

Supplement: Figure S8 — Inhibition of thrombin induced fibrin clotting with varying concentrations of free GPR and GPS peptides. Only at high concentrations (≥ ∼ 300 µM) of GPR peptide was an effect observed. The concentrations used in Figure 3b of the main text (10–60 µM, starred on graph) showed no effect for either peptide. (TIFF) [file pone.0100678.s008.tiff]

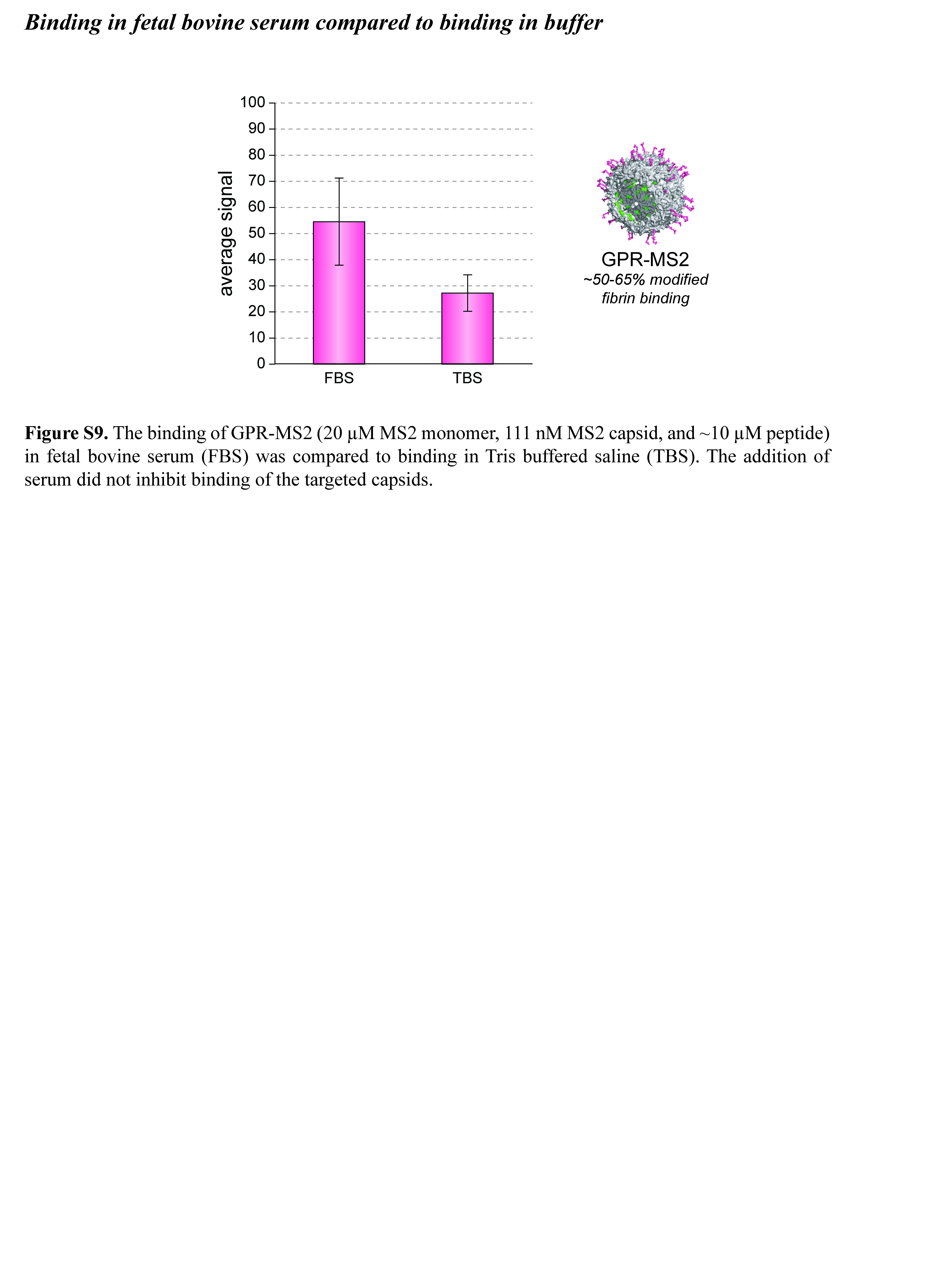

Supplement: Figure S9 — The binding of GPR-MS2 (20 µM MS2 monomer, 111 nM MS2 capsid, and ∼10 µM peptide) in fetal bovine serum (FBS) was compared to binding in Tris buffered saline (TBS). The addition of serum did not inhibit binding of the targeted capsids. (TIFF) [file pone.0100678.s009.tiff]

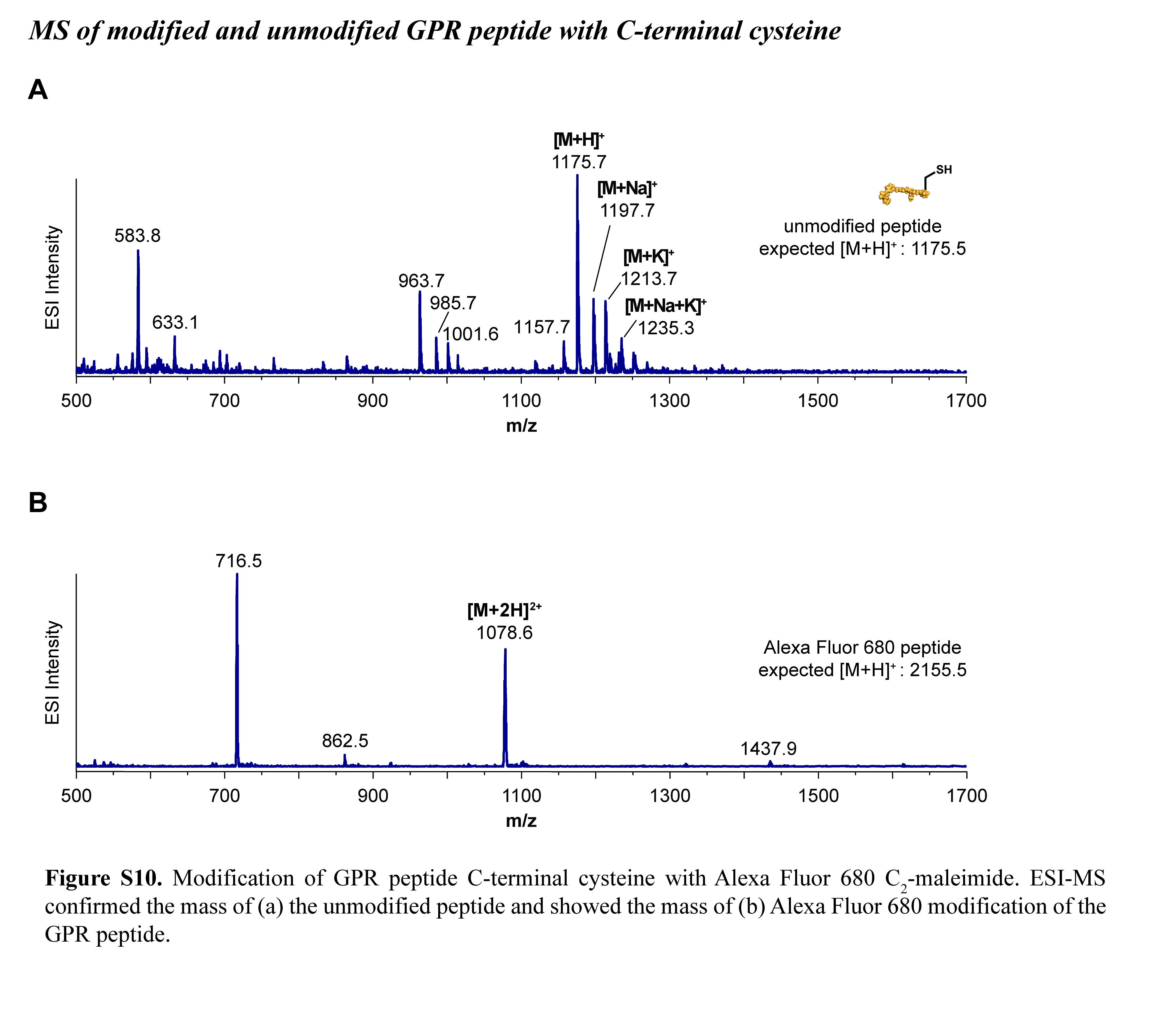

Supplement: Figure S10 — Modification of GPR peptide C-terminal cysteine with Alexa Fluor 680 C2-maleimide. ESI-MS confirmed the mass of (a) the unmodified peptide and showed the mass of (b) Alexa Fluor 680 modification of the GPR peptide. (TIFF) [file pone.0100678.s010.tiff]

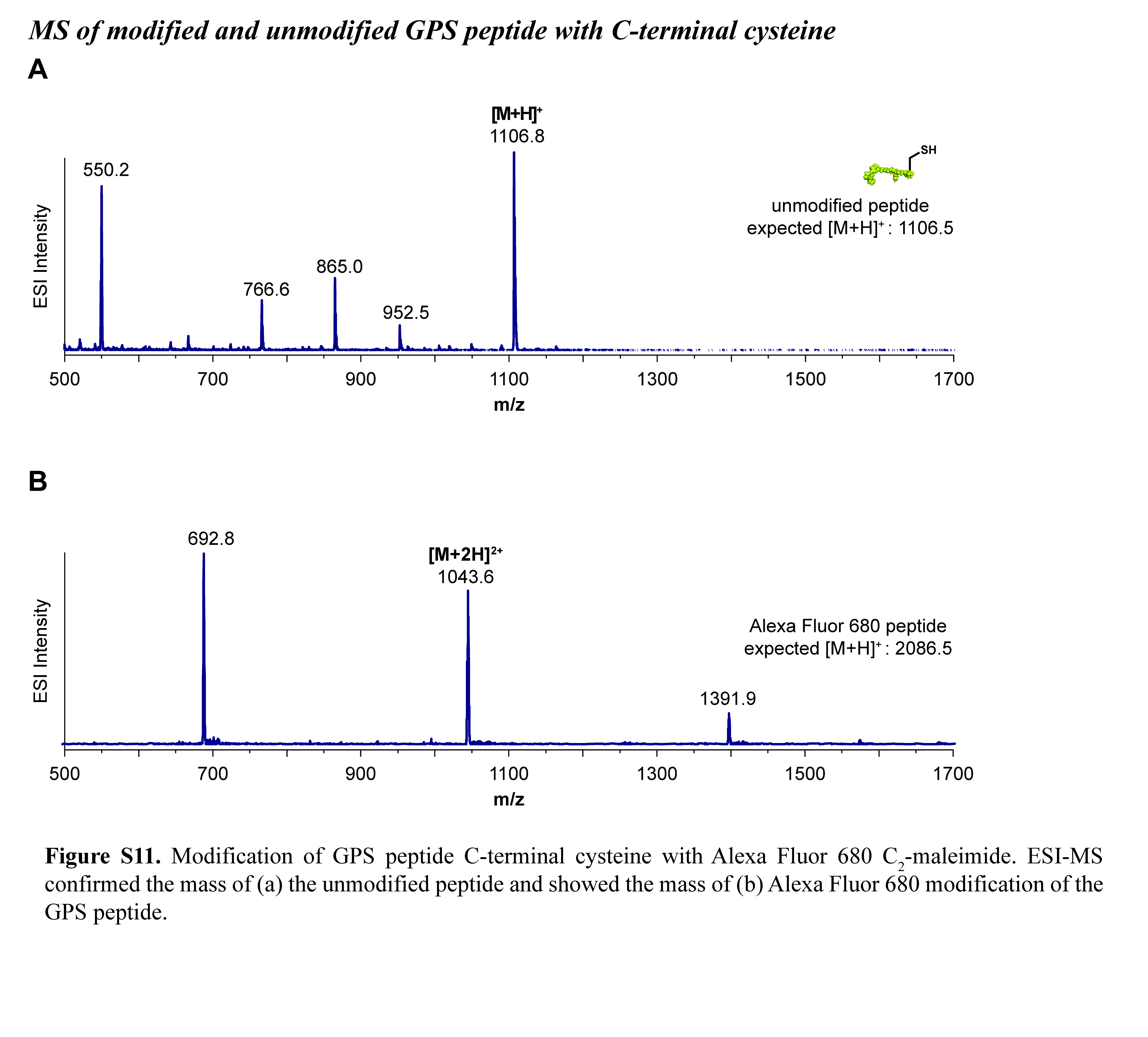

Supplement: Figure S11 — Modification of GPS peptide C-terminal cysteine with Alexa Fluor 680 C2-maleimide. ESI-MS confirmed the mass of (a) the unmodified peptide and showed the mass of (b) Alexa Fluor 680 modification of the GPS peptide. (TIFF) [file pone.0100678.s011.tif]

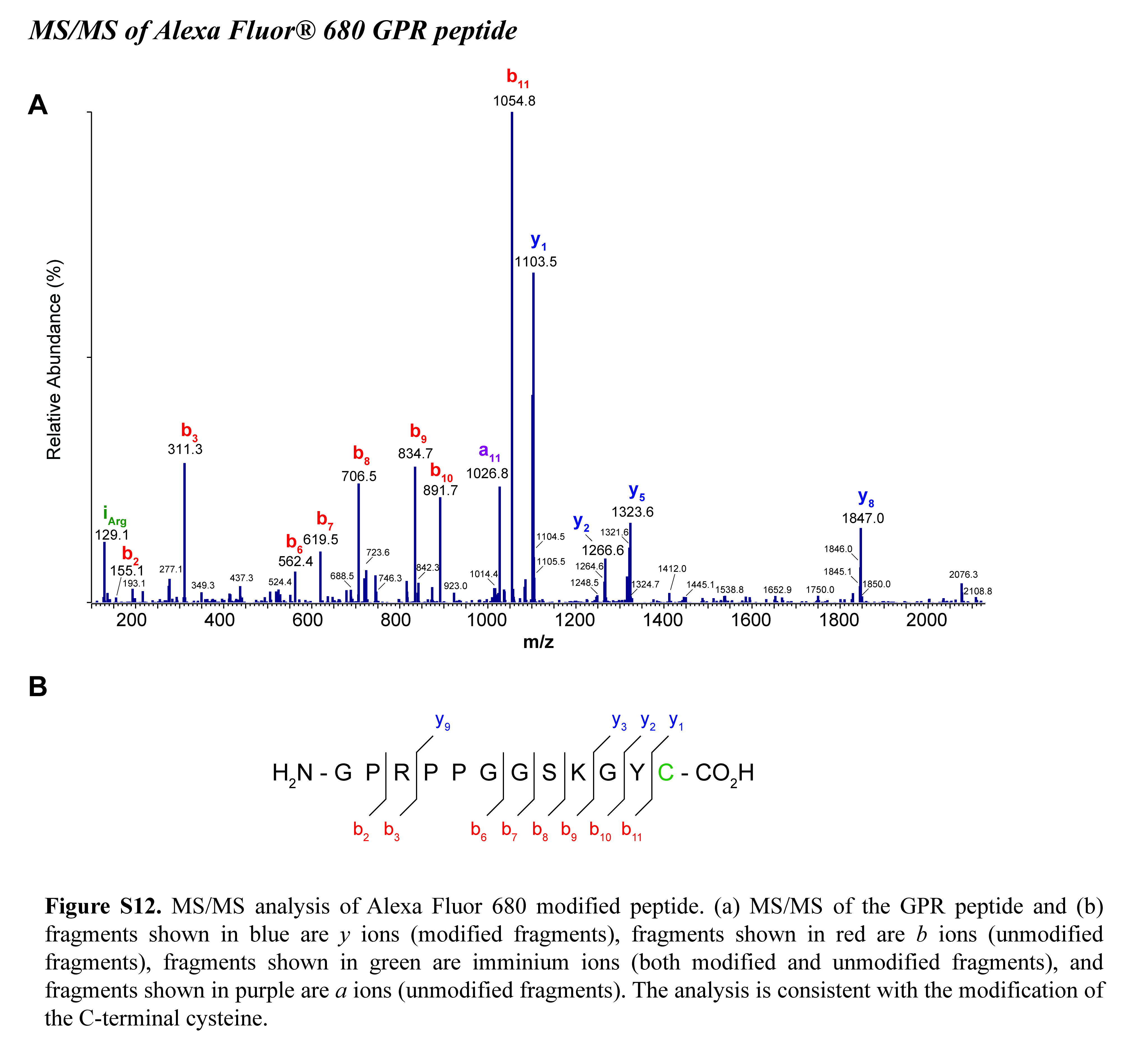

Supplement: Figure S12 — MS/MS analysis of Alexa Fluor 680 modified peptide. (a) MS/MS of the GPR peptide and (b) fragments shown in blue are y ions (modified fragments), fragments shown in red are b ions (unmodified fragments), fragments shown in green are imminium ions (both modified and unmodified fragments), and fragments shown in purple are a ions (unmodified fragments). The analysis is consistent with the modification of the C-terminal cysteine. (TIFF) [file pone.0100678.s012.tiff]

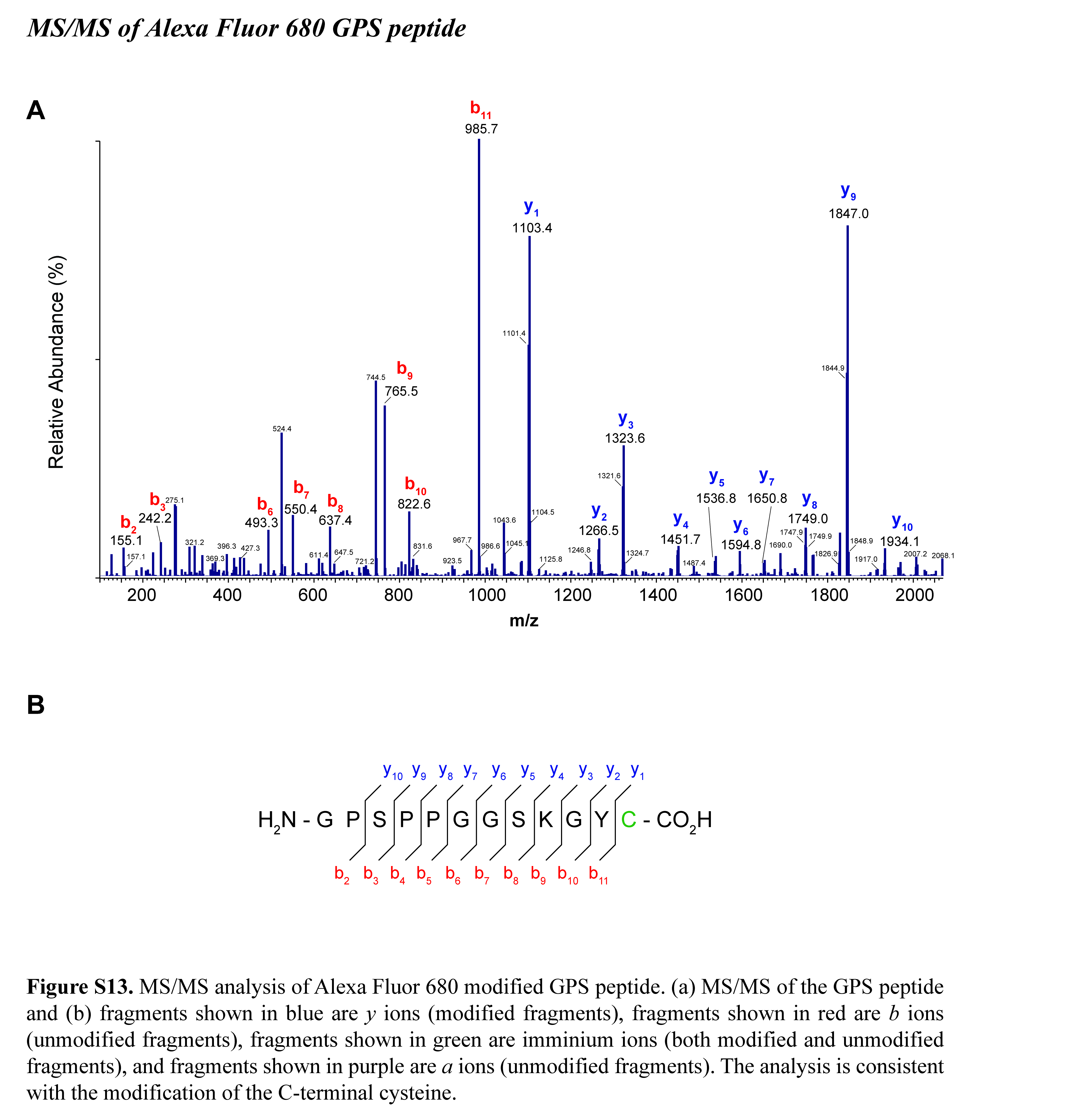

Supplement: Figure S13 — MS/MS analysis of Alexa Fluor 680 modified GPS peptide. (a) MS/MS of the GPS peptide and (b) fragments shown in blue are y ions (modified fragments), fragments shown in red are b ions (unmodified fragments), fragments shown in green are imminium ions (both modified and unmodified fragments), and fragments shown in purple are a ions (unmodified fragments). The analysis is consistent with the modification of the C-terminal cysteine. (TIFF) [file pone.0100678.s013.tiff]

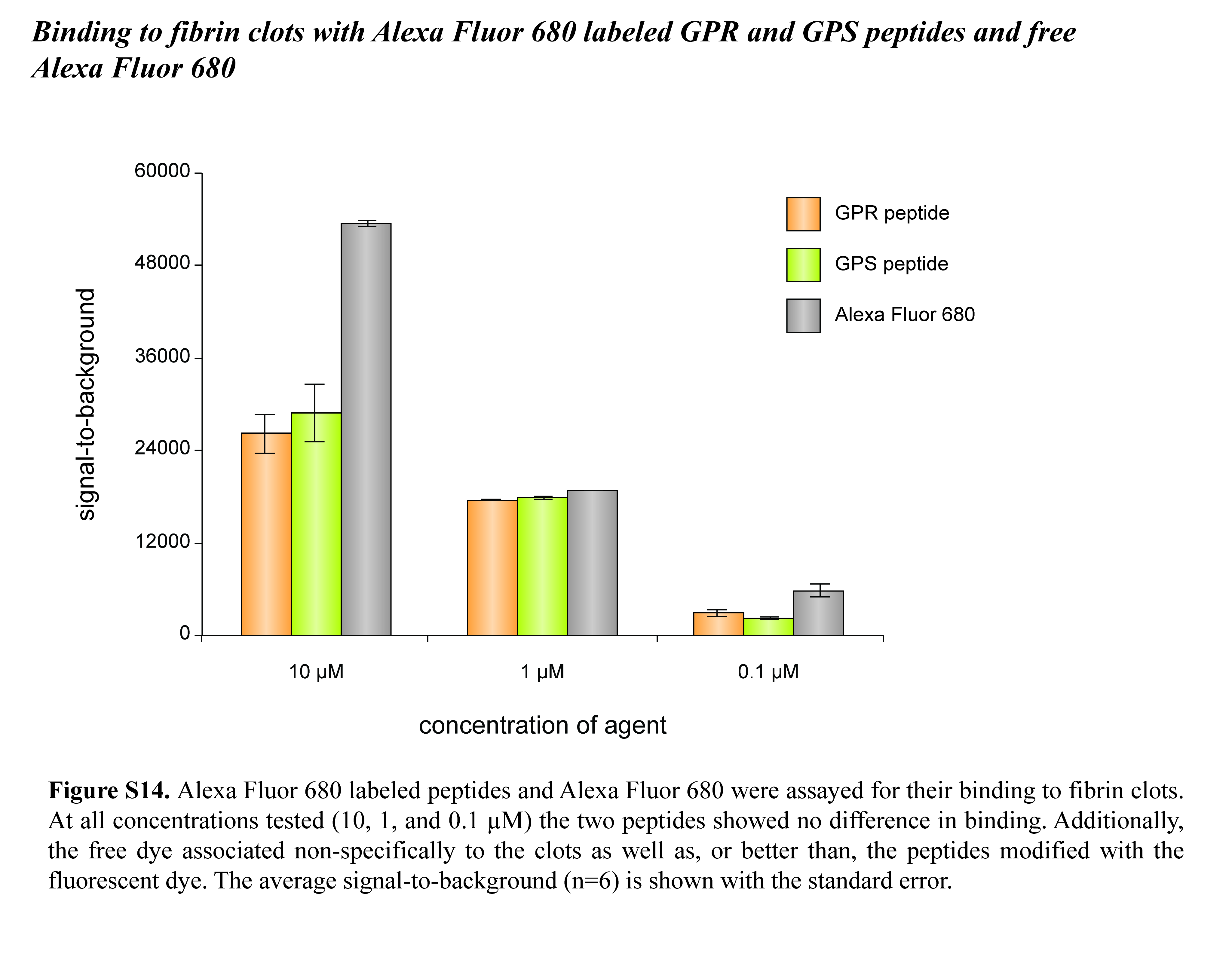

Supplement: Figure S14 — Alexa Fluor 680 labeled peptides and Alexa Fluor 680 were assayed for their binding to fibrin clots. At all concentrations tested (10, 1, and 0.1 µM) the two peptides showed no difference in binding. Additionally, the free dye associated non-specifically to the clots as well as, or better than, the peptides modified with the fluorescent dye. The average signal-to-background (n = 6) is shown with the standard error. (TIFF) [file pone.0100678.s014.tiff]
